# Deep Learning Reaction Framework (DLRN) for kinetic modeling of time-resolved data

**DOI:** 10.1038/s42004-025-01541-y

**Published:** 2025-05-15

**Authors:** Nicolò Alagna, Brigitta Dúzs, Vincent Dietrich, Ali Tayefeh Younesi, Livia Lehmann, Ronald Ulbricht, Heinz Köppl, Andreas Walther, Susanne Gerber

**Affiliations:** 1 Institute of Human Genetics, University Medical Center, Mainz, Germany; 2 Department of Chemistry, Johannes Gutenberg University, Mainz, Germany; 3 Max-Planck Institute for Polymer research, Mainz, Germany; 4 Centre for Synthetic Biology, Technische Universität Darmstadt, Darmstadt, Germany

**Keywords:** Reaction kinetics and dynamics, Computational chemistry

## Abstract

Model-based analysis is essential for extracting information about chemical reaction kinetics in full detail from time-resolved data sets. This approach combines experimental hypotheses with mathematical and physical models, enabling a concise description of complex system dynamics and the extraction of kinetic parameters like kinetic pathways, time constants, and species amplitudes. However, building the final kinetic model requires several intermediate steps, including testing various assumptions and models across multiple experiments. In complex cases, some intermediate states may be unknown and are often simplified. This approach requires expertise in modeling and data comprehension, as poor decisions at any stage during data analysis can lead to an incorrect kinetic model, resulting in inaccurate results. Here, we introduce DLRN, a new deep learning-based framework, designed to rapidly provide a kinetic reaction network, time constants, and amplitude for the system, with comparable performance and, in part, even better than a classical fitting analysis. We demonstrate DLRN’s utility in analyzing multiple timescales datasets with complex kinetics, different 2D systems such as time-resolved spectra and agarose gel electrophoresis data, experimental datasets as nitrogen vacancy and strand displacement circuit (using photoluminescence and transient absorption techniques), even in scenarios where the initial state is a hidden, non-emitting dark state.

## Abstract (web summary)

Building kinetic models from time-resolved data can be challenging due to the presence of unknown intermediate states. Here, the authors introduce a deep- learning-based framework that provides a kinetic reaction network and outperforms conventional fitting in determining time constants and amplitude parameters.

## Introduction

Time-resolved techniques, such as spectroscopy, chromatography or microscopy, are powerful and widely used tools for studying and investigating the kinetics of chemical reactions in complex systems in biology, chemistry, and physics^1–5^. These techniques generate one-, two-, or three-dimensional data sets, in which changes in the signal intensity are tracked as a function of time, which is one of the independent experimental variables. For example, time-resolved spectroscopy measurements are composed of two independent experimental variables, one being related, for example, to wavelength or magnetic field strength. The other is the probing time after excitation, which is used to track changes in the signal during the measurement. To reveal the processes underlying the observable changes in signal, computational and mathematical methods are necessary to analyze mechanistic details of reactions or decipher networks of chemical reactions, and, ultimately extrapolate the molecular properties linked to the evolution of a specific system in question^5,6^.

The necessity of introducing mathematical methods arises from the fact that short-lived intermediate species can be difficult to detect with experimental measurements^7,8^. Indeed, many cases require combinations of different experimental techniques to identify specific reaction pathways. Furthermore, the chemical, biochemical, and cellular processes of complex systems require theoretical modeling to effectively disentangle information regarding their dynamics^9–14^. In practice, working with mathematical models for kinetic studies is almost mandatory, and the strategies used for data analysis can be classified into two families: model-independent and model dependent^5,15–18^.

For instance, in photochemistry, model-independent analysis is usually a global fitting technique (or global analysis, GA), which consists of a simultaneous analysis of multiple kinetic traces at different wavelengths using exponential functions. This method provides a functional description of a kinetic profile of a system, extrapolating the decay-associated amplitude *A*_0_(*λ*) for each exponential function, primarily known in spectroscopy as decay-associated spectra (DAS), and the minimum number of time constants *τ* involved in the mechanism. Although GA is usually a necessary step in studying time-resolved systems, it is only an initial step in the analysis because it provides only partial information about the dynamics. The combination of a GA with the photochemical model is usually called global target analysis (GTA)^5,15,19^, which falls into the model-dependent category. This analysis is essential because kinetic modeling can quantitatively extrapolate kinetic mechanisms from time-resolved data, providing kinetic parameters such as species-associated amplitude (or species-associated spectra, SAS) and decay-time constants to describe complex photochemical dynamics.

Although GTA is a well-established method in which each species is connected to another via differential equations, this analysis is not always straightforward since it involves devising and testing kinetic reaction models based on previous knowledge and selected assumptions^5,15,18,20–22^. This can lead to an increase in the minimum number of parameters previously found by GA and generation of a set of models of which the most reasonable must be determined. This points to the challenge of using GTA to identify the correct kinetic model by the interplay between data and modeling approaches. Such problems scale increasingly poorly if the system becomes complex and has many variables to compute. For example, ATP-driven DNA dynamics^23–26^ show multi-step strand formation controlled by several enzymatic reactions. The kinetic model is challenging to determine, and the analysis requires the accurate selection and combination of chemical engineering and modeling approaches.

In recent years, the scientific community has increasingly worked on finding ways to study and describe chemical reaction networks (CRNs) using automated computational methods^27–30^. In particular, the application of machine learning (ML) methods to chemical reactions has significantly increased in popularity^6,28,31–44^. In this growing field of research, neural network models are trained with synthetic data and already highly validated data to set the ground truth of the system such that the neural network learns to extrapolate complex patterns in the data through supervised learning. This capability of supervised neural networks to extract complex information from data is validated by comparing the network’s predictions, which are the outputs of the ML model after data processing, with expected results derived from unseen data with a known ground truth, making it possible both to evaluate the performance and accuracy of the network and its ability to analyze the system correctly. These methods are not only used for analyzing chemical reactions of known and new chemical compounds^45–51^, but have also been applied to the study of proteins^52–55^ to probe the free-energy and protein conformational landscape for extrapolating key features of biological systems.

This work presents DLRN (Deep Learning Reaction Network), a deep neural network based on an Inception-Resnet^56^ architecture created to combine GTA and ML methods. DLRN can disentangle all the kinetic information from a 2D time-resolved data set, giving the most probable kinetic model, related time constants for each pathway, and a maximum of four SAS for one timescale. Moreover, DLRN can analyze systems in which the initial state is a hidden state. We also tested DLRN to analyze 2D data at multiple time scales, achieving good performance and the ability to identify complex kinetic models that have a higher number of variables (but not for each timescale) than those used during training. DLRN performed well in the analysis of both time-resolved spectra and electrophoresis images. Moreover, DLRN was also tested to analyze the kinetic reaction of nitrogen-vacancy (NV) centers and DNA strand displacement (DSD) networks measured using various experimental techniques like photoluminescence (Pl) and transient absorption (TA). We show that the neural network was able to extract the correct expected kinetic models, including their variables, for these very different experimental systems as analyzed by various techniques and data sets of different dimensionalities.

## Results

### DLRN analysis of synthetic time-resolved spectral data

**Fig. 1:**
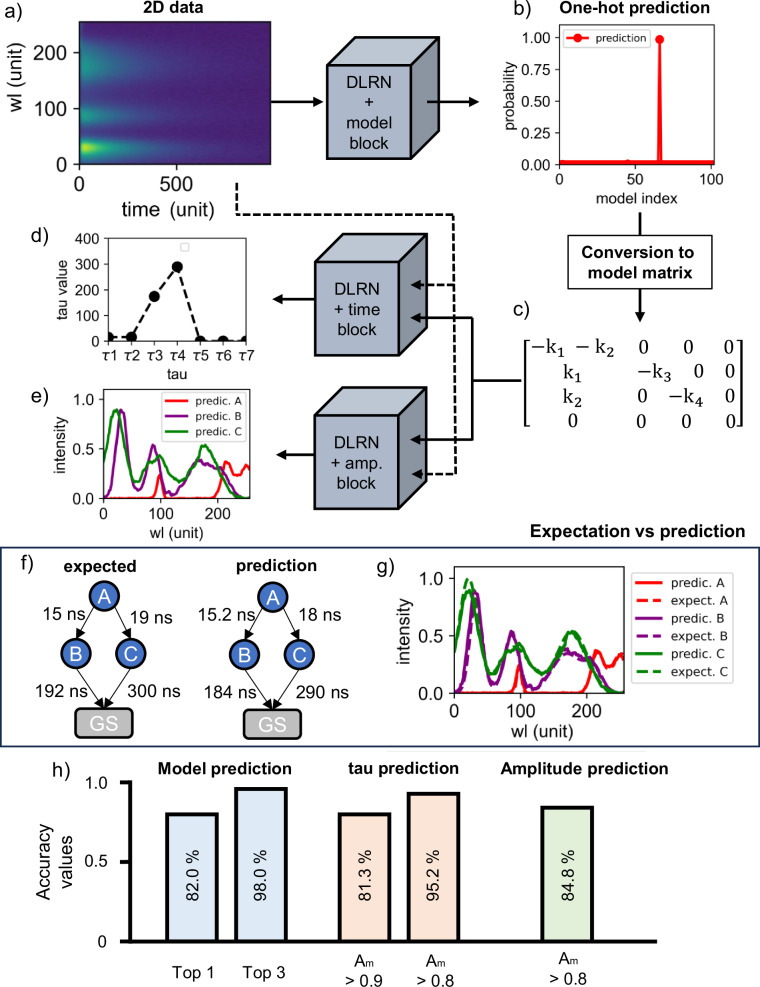
DLRN work-flow analysis of 2D images. **a** Example of 2D spectra data used for the analysis. **b** One-hot encoding prediction obtained from DLRN using the model block. Each position represents a specific kinetic model. **c** Conversion of the one-hot encoding prediction into a model matrix. This model matrix is used in both time and amplitude blocks for predicting time constants and amplitudes. **d** Time constant predictions obtained from DLRN using the time block. **e** Normalized amplitude prediction obtained from DLRN using the amplitude block. **f** Comparison between expected and predicted models with the corresponding time constant for each decay pathway. **g** Comparison between expected and predicted spectra. The predictions of model, time constants, and amplitudes match well the expected results. **h** Accuracy values for model, tau and amplitude prediction obtained using DLRN on 100k unknown 2D datasets (See also Table SI 1).

DLRN has an Inception-Resent like architecture^56^, where personalized features and blocks (Time, Amplitude and Model) were designed to analyze time-resolved data (see Methods and SI section 1). Figure 1 shows the entire DLRN pipeline for analyzing a time-resolved data set. The data set (Fig. 1a), representing a 2D image containing wavelength and time information, is sent through the neural network and analyzed using the model block computational pathway. This block analyzes the 2D signal, giving a one-hot encoding representation of the most probable model that DLRN can predict from 102 different models (Fig. 1b). Next, the one-hot encoding prediction is converted into the model matrix (Fig. 1c). An explanation of this representation of the kinetic model is given in Supplementary note 2. The model matrix, which provides information about the possible rate constants and pathways involved, is transformed into a binary matrix, where each *k*_i_ is substituted by a token of value 1, but without changing the signs in front of the rate constants. This passage is essential as the model prediction should operate for both time constants (*τ*) and wavelength amplitude (wl) analysis. The binary matrix is then sent as a second input for the DLRN time and amplitude blocks, which can extrapolate the numbers and values for the corresponding time constants (Fig. 1d) and amplitudes (Fig. 1e) linked to the kinetic model of the data set analyzed. DLRN can extrapolate up to seven *τ* values and up to four spectra. The efficiency of the DLRN is illustrated by a comparison between the expected and predicted kinetic models (Fig. 1f), which shows the chemical reaction network (CRN) and time constant values for each decay pathway in both cases. DLRN can predict the correct kinetic model with high-probability confidence (Fig. 1b) and with good approximation, accurate time constants for each decay path. Furthermore, a comparison between expected and DLRN-predicted amplitudes shows again a good agreement between measured and simulated values (Fig. 1g). Further comparisons between DLRN predictions and expected values are given in Supplementary note 3.

To quantify the network performance in a larger number of samples, we tested DLRN on an evaluation batch containing 100,000 unknown 2D data sets. Figure 1h and Table SI 1 shows the accuracy values obtained for the model, time constants, and amplitude predictions. We used the area metric *A*_m_ (see Methods, Area metric for regression accuracy) to quantify the accuracy of the regression analysis on the amplitudes and time constants. The test revealed that in 83.1% of cases (Top 1 in Table SI 1), the DLRN correctly predicted the expected model (with a prediction confidence of >95%), whereas in 14.9% of cases (which adds up to 98.0% for Top 3 in Table SI 1), one of the three most likely predictions matched the expected kinetic model. Specifically, the Top 1 accuracy counts how often DLRN prediction and expectation have an exact match, while the Top 3 accuracy indicates when one of the three most probable predictions matches the ground truth. This shows that DLRN predicted the kinetic model of time-resolved spectral data sets with high confidence. For predicting time constants on the evaluation batch, DLRN accuracy was 80.8% using *A*_m_ > 0.9, which means that the forecasts have an average error of less than 10% of the expected values. If we reduced *A*_m_ > 0.8 (error less than 20%), DLRN accuracy increases to 95.2%, which means that DLRN almost always predicted time constants with an error of no more than 20%. High performance was also shown for the prediction of amplitude for the evaluation batch by DLRN. Using *A*_m_ > 0.8, the DLRN accuracy was 81.4%. However, since DLRN extrapolates four spectra during the analysis for the amplitude prediction, the error limit (20%) is the sum of the errors of these four spectra, indicating that each spectrum is subject to an error of less than 5% compared to the expected outcome.

Next, we wanted to test whether DLRN can also be used to analyze time-resolved data sets in which the initial state is a dark state (or hidden state), i.e., one that is involved in the kinetic reaction, but it is not directly visible in the data. Figure SI 7 shows DLRN predictions compared to the expected results. The DLRN analysis shows good agreement with the expected kinetic model, including time constant values and species amplitudes. Notably, the amplitude for the initial state A is equal to zero, indicating that DLRN can understand when the initial state is “off”, but that it can also extrapolate the correct kinetic model. In this case, DLRN was also tested on an evaluation batch of 100,000 unknown 2D data sets. The results were similar to those shown in Table SI 1 (see Supplementary note 3).

### Comparing DLRN with existing fitting methods

**Fig. 2:**
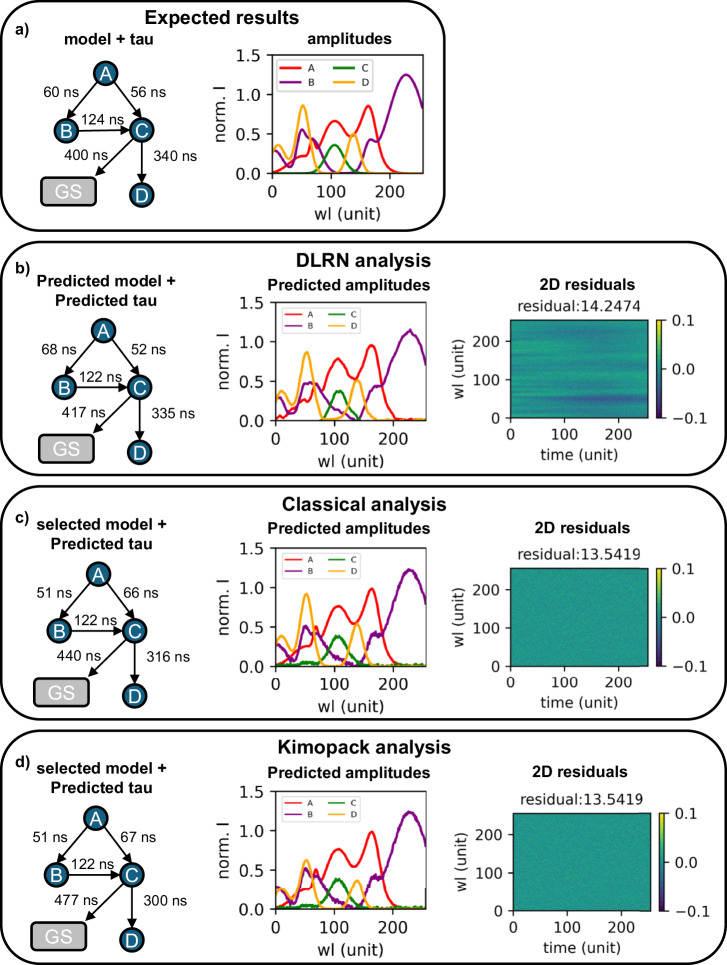
Comparison between DLRN and classical analysis used on the same complex molecular dynamics. **a** Expected kinetic model with time constants for each decay pathway (left) and the respective spectra for each species (right). **b** Kinetic model pathways and time constants (left), spectra for each species (middle), and residuals (right) obtained by DLRN analysis. **c** Kinetic model pathways and time constants (left), spectra for each species (middle), and residuals (right) obtained by classical fitting analysis. **d** Kinetic model pathways and time constants (left), spectra for each species (middle), and residuals (right) obtained by KiMoPack analysis.

The performance of the DLRN neural network was compared to the classical fitting analysis and KiMoPack^57^, which are two common algorithms used for time-resolved analysis. Figure 2a shows the expected model and relative spectra for the 2D data set used for the comparison. The time-resolved data set presents a complex mechanism, which is challenging to analyze. Starting with the model prediction, DLRN can identify the correct model with a probability confidence of 98.9% (Fig. 2b). Moreover, the time constants predicted by the neural network match the expected one with good approximation, including the branching ratio (compare modeled time constants in Fig. 2a, b). Additionally, DLRN predicts accurately the amplitudes (Fig. 2b and Fig. SI 8d), showing that it can also extrapolate the correct spectra for complex dynamics. The residuals between the DLRN analysis and original data (Fig. 2b) show some small residual contribution, indicative of minor differences with the original signal. Figure 2c shows the classical fitting analysis performed on the same datasets. To use a classical analysis, it is necessary to define the kinetic model for the fit, whereas DLRN can extrapolate the model by itself. When the correct model was imposed for classical analysis, the time constants and the amplitudes were free to change during the fitting for extrapolation. Starting with the time constants (Fig. 2c), the fit analysis shows a mismatch in the tau values, especially in the branching pathways of A (compare predicted taus in Fig. 2a, c). Moreover, the amplitudes of some spectra show several mismatches compared to the expected one (compare predicted amplitudes in Fig. 2a, b). To emphasize the differences, we subtracted the predicted spectra to the expected spectra, showing clear discrepancies between them (Fig. SI 8e).

This mismatch can be attributed to the compensation of time constants during the fitting, indicating that a local minimum was reached during the analysis. Residuals of the fit analysis (Fig. 2b) are better than those of DLRN (Fig. 2c). We also investigate amplitude prediction in simplest cases (linear mechanism), find that DLRN analysis and classical fit analysis converge, on average, to similar or even the same results (Fig. SI 7). We further used KiMoPack to analyze the same data and compare the outcome with those of DLRN. Similar to the classical fit, KiMoPack needs a selected kinetic model before the analysis. Choosing the exact expected model for KiMoPack, the time constants and the amplitudes were free to change during the fitting for extrapolation. KiMoPack results show a similar trend compared to the classical fit method, where mismatches between expected and predicted values were observed in both time constant values (especially for the branch pathway of the state A) and amplitudes intensity (compare Fig. 2a, d and SI Fig. 8). However, KiMoPack shows a better spectrum prediction compared to the classical fit method (Fig. 2d and Fig. SI 8f). Also in this case, KiMoPack (Fig. 2d) residuals are better than those of DLRN (Fig. 2c). The results presented in this section show that DLRN can perform at least as well as other time-resolved analysis methods and, in some cases, even better without any model assumption. It can obtain the correct minimum solution and automatically extrapolate all parameters for the analysis in a few passes. This allows fast and accurate computation of time-resolved data.

### DLRN analysis on multi-time scale and complex model systems

**Fig. 3:**
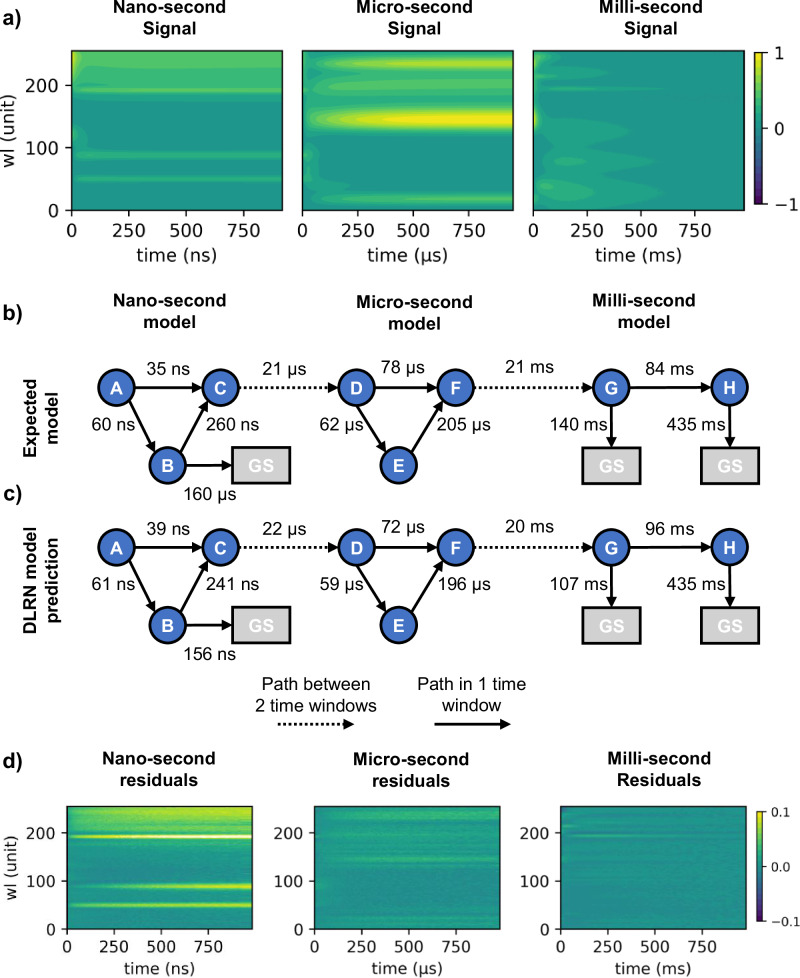
DLRN analysis of a complex and multi-time scale system. **a** Nanosecond (left), microsecond (middle) and millisecond (right) time resolved signal analyzed with DLRN. **b** Expected kinetic model with decay time constants for each pathway. **c** Predicted kinetic model using DLRN. predicted time constants are shown for each decay pathway. **d** Residual between original signal and DLRN fit.

DLRN was created to analyze a kinetic model that can involve a maximum combination of four active state (plus a ground state -GS-) in one time scale. The purpose of creating DLRN was to have a neural network capable of analyzing reasonably complex systems in a “time unit” or “time window”. In this section, we want to test the limit of DLRN by analyzing a kinetic model that involves more than four active states, but in three time scale (e.g., from nanosecond to millisecond time scale). To use DLRN for the analysis of this more complex mechanism, it is necessary to split the dataset into three samples, one for each time scale (Fig. 3a), and preprocess each subset individually. Preprocessing includes: i) transformation of the original time scale between 0 and 1000; ii) signal normalization for the maximum absolute intensity value; iii) interpolation of the original signal for the same time scale used to train DLRN. As said before, this is necessary because DLRN was projected to analyze one time scale at a time. After using DLRN for the analysis of each individual time scale, the results were interpreted and re-combined back to recreate the full dynamics and the results were compared with the original expected kinetic model (Fig. 3b, c). The expected model shows a complex dynamic involving a total amount of eight active state, where three of them show a decay path to the ground state (Fig. 3b). Using DLRN, we were able to reconstruct the complex expected kinetic model (Fig. 3c). It was able to recognize the complex path in each timescale, with good approximation of the time constant values with an error, on average, of about 6% (compare Fig. 3b with Fig. 3c). The dashed arrows show kinetic path that could be observed in two datasets due to long excited state transition. However, the correct time constant value was always obtained from the DLRN analysis on the most suitable time window. For example, to analyze the C → D decay pathway, both the nanosecond and microsecond time scales show this transition, which helps the interpretation of the results obtained by DLRN. However, the correct time constant ${\tau }_{C\to D}$ was obtained from the DLRN microsecond analysis. We also analyzed the residuals between expected signal and DLRN fitting (Fig. 3d). The residuals still show some artifact of the signal, which can be linked to partial misprediction of the time constants and/or amplitudes values. However, we observed before that DLRN tries to obtain the best predictions without compensation, while other methods try to obtain the best residuals by incorrectly predicting the real values (see for example Fig. 2). A second multi-scale complex model was analyzed using DLRN and results are shown in Fig. SI 20. Despite DLRN could be used in several scenarios with good approximate predictions, additional work to improve neural network residuals is necessary for a better interpretation of future results. We also investigate how time step (Δt), or granularity, can affect the neural network performances. This is important because the data is interpolated to match the neural network Δt used during the training. We analyzed five different Δt values (0.5, 1, 2, 10 and 20) and for each of them we created 20k data set and then screened the DLRN performances (Fig. SI 21). The analysis showed that model, time constants and amplitudes prediction remain quite consistent for Δ*t* ≤ 2, while DLRN performances reduction start to be visible for Δ*t* = 10. Considerable reductions were observed for Δ*t* = 20. However, we associate the reduction in DLRN performance with the fact that a large part of the initial dynamics is lost during interpolation when large Δ*t* is used. This is because DLRN is trained with a combination of linear (for initial dynamics) + exponential (for long dynamics) time scale, which means that large Δ*t* will not correctly represent the initial dynamics but only the long one, generating loss of information.

### Expanding DLRN analysis on synthetic time-resolved agarose gel electrophoresis and transient absorption spectroscopy

**Fig. 4:**
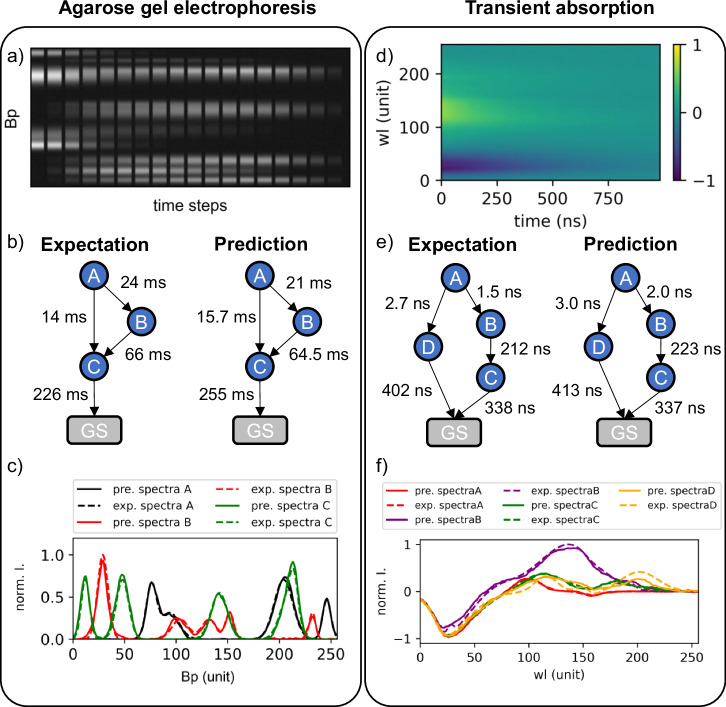
DLRN analysis of 2D time-resolved agarose gel electrophoresis and time resolved transient absorption. **a** Example of a synthetic agarose gel used for the kinetic model analysis with DLRN. **b** Expected model and DLRN analysis performed on the image shown in panel a. DLRN can correctly predict both the kinetic model and the time constant associated with each pathway. **c** Comparison between DLRN prediction (continuous lines) and expected (dashed lines) amplitude from the image in **a**. **d** Example of transient absorption signal used for the DLRN analysis. **e** Expected model and DLRN analysis performed on the image shown in panel **d**. DLRN predicts correctly the model and time constants within a small error. **f** Comparison between DLRN prediction continuous lines) and expected (dashed lines) amplitude from the image in **d**.

DLRN was then trained to analyze synthetic images of both time-resolved agarose gels and time-resolved transient absorption spectroscopy signal (TA). Starting with gels, they are used to track molecular changes at different time delays e.g., in ATP-driven DNA-based reaction networks^26^, and we were interested in using DLRN to obtain and investigate the molecular dynamics that control such systems. The synthetic agarose gel images were generated similarly to the spectral images (see Methods, Generation of synthetic data for training and evaluation), with the difference that the gels had a maximum number of 17 time points (the possible cells of the gel), which means that each time point covers more pixels in the x-axis. The base pair (Bp) spectrum of each state was generated by summing a random number of one to eight Gaussian functions with random peak position, width, and intensity. Figure 4a–c shows an example of a simulated time-resolved agarose gel and DLRN analysis. Also in this case, DLRN was tested on an evaluation batch containing 100,000 unknown 2D data of time-resolved agarose gel. Table SI 2 (Supplementary note 3) shows the accuracy values obtained for the model, time constants, and amplitude predictions. DLRN model prediction had an accuracy of 82.4% for the Top 1, while in 16.2% of cases, i.e., a total accuracy of 98.2% for the Top 3, one of the three most likely predictions obtained from DLRN matches the expected kinetic model. DLRN showed good performance in predicting time constants. In 72.8% of cases (*A*_m_ ≥ 0.9; Table SI 2), we obtained an error of less than 10%, while in 92.1% of the predictions (*A*_m_ > 0.8; Table SI 2), the error was less than 20%. The amplitudes of agarose gel spectra were also analyzed using DLRN. Here, predictions of individual amplitudes differed on average by no more than 7% from the real spectra in 76% of the batch dataset. We observed that this percentage could be increased to 83.1% by adding a dilation filter (=3) in all the separable convolution 1D layers in the second residual block (see amplitude block, Fig. SI 2). These high-performance results show that DLRN can also be used to analyze time-resolved agarose gels for tracking dynamics during molecular assembly. Further DLRN analysis and results of time-resolved agarose gels are reported in Supplementary note 5.

Besides agarose gels, DLRN was also trained to analyze synthetic time resolved TA signals to obtain and investigate kinetic reaction tracked by this time-resolved spectroscopy technique. As before, the synthetic TA images were generated similarly to the emission spectral images (see Methods, Generation of synthetic data for training and evaluation), but with the difference that a negative signal (Ground state bleach + stimulated emission) was added to each spectrum to simulate the *ΔA* tracked during TA measurements. Figure 4d–f shows an example of DLRN analysis on TA signal. As before, DLRN TA analysis was tested on an evaluation batch containing 100,000 unknown 2D data of time-resolved TA signal and accuracy values obtained for model, time constants and amplitude predictions are shown in Table SI 3. DLRN Top 1 model prediction shows an accuracy value of 87%, while in the 11.5% of the cases (summing up at 98.5%) one of the Top 3 solutions matches the exact model. DLRN time constants prediction also shows good accuracy values of 81.7% (*A*_m_ ≥ 0.9; Table SI 3) having an error below 10%, while in 96.6% of the predictions (*A*_m_ > 0.8; Table SI 3), the error was less than 20%. Here, predictions of individual amplitudes differed on average by no more than 5% from the real spectra in 83.1% of the batch dataset.

### DLRN analysis on DNA strand displacement networks

**Fig. 5:**
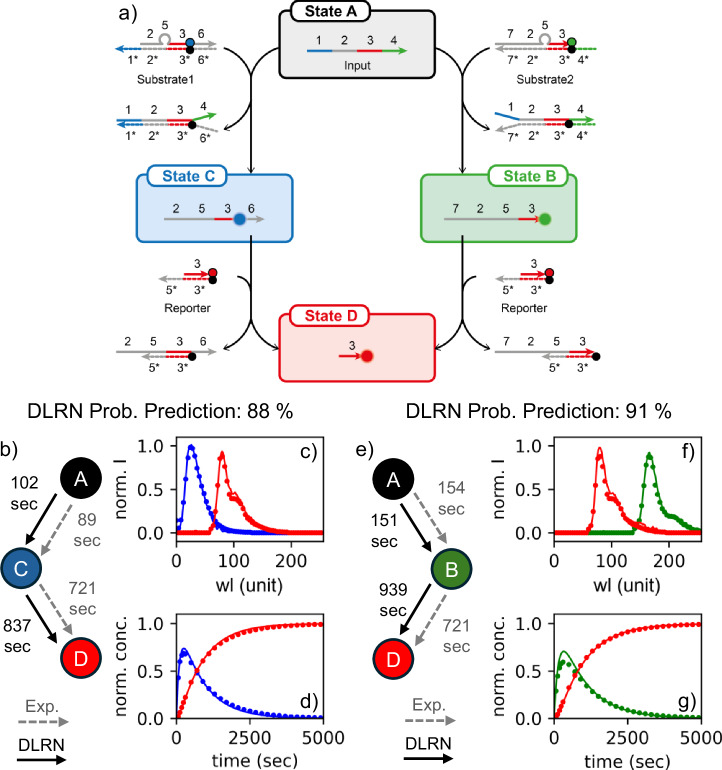
DSD kinetic reaction analysis for Su1- and Su2-only dynamics. **a**, General DSD kinetic reaction mechanism. Region 1 (blue continue line in the input vector of state A) is the toehold. **b**, **e** Comparison between DLRN (continuous black arrows) and expected (dashed gray arrows). Kinetic models for Su1 only (**b**) and Su2 only (**e**) with time constants shown for each decay pathway. The initial state A is a dark state. **c**, **f** Expected (filled circles) and DLRN-predicted (continuous line) spectra for Su1 only (**c**) and Su2 only (**f**) dynamics. **d**, **g** Expected (filled circles) and DLRN-predicted (continuous lines) kinetic traces for Su1 only (**d**) and Su2 only (**g**) dynamics. Colors correspond to the kinetic model.

We used DLRN to analyze and disentangle DNA strand displacement (DSD) CRNs. DSD CRNs provides an efficient toolbox with many design options (network architecture, reaction rates) to create complex nonlinear behavior in life-like materials systems based on the strict rules of DNA base-pairing^58^. The kinetics of simple DNA strand interactions can be predicted very well numerically (e.g., using the Visual DSD program^59^). However, these numerical predictions are no longer realistic if enzymatic reaction steps, pH-dependent conformational changes, or the presence of dye and quencher molecules on the DNA strands affect the kinetics of DSD. In these cases, DLRN analysis is necessary to understand the dynamics of a complex DSD CRN. Here, DSD dynamics involve four different DNA strands (Fig. 5a), denoted input (Inp), substrate 1 (Su1), substrate 2 (Su2), and reporter (Re). The mechanism of DSD is a branching system in which Inp (state A) can interact with Su1 and Su2, liberating two different fluorescent strands that correspond to states B and C. Then, these two strands can react with Re, and both liberate the same last fluorescent DNA strand (state D). We designed versions of the CRN with different sequences (sequence fragment 1 in blue of Su1, Fig. 5a) to tune the reaction rate of branch C. The corresponding DNA sequence is given in Supplementary Note 8. Limited to pseudo-first-order reactions, DLRN was initially used to analyze the simple cases in which one of the branching pathways was turned off by using only one substrate at a time. Data generation is described in detail in the Methods section. Figure 5 shows the comparison between the DLRN analysis and the expected model, spectra, and kinetic traces obtained by Visual DSD simulations only if Su1 = 20 nM (Fig. 5b–d) or Su2 = 20 nM (Fig. 5e–g); Inp and Re concentrations were constant at 10 and 30 nM, respectively. In both cases, DLRN was able to correctly identify the CRN (Fig. 5b, e), extrapolate the correct spectral amplitudes involved (Fig. 5c, f), and reconstruct the kinetic traces (Fig. 5d, g). Although expected (Visual DSD) and predicted (DLRN) kinetic traces overlap with each other, the values of ${\tau }_{C\to D}$ and ${\tau }_{B\to D}$ differed significantly from the expected values (in particular, the ${\tau }_{B\to D}$ value differed by about 35%), while the values of ${\tau }_{A\to B}$ and ${\tau }_{A\to C}$ match the values with an error of less than 10%. Due to this discrepancy, we created the kinetic traces for both the dynamics imposing for ${\tau }_{C\to D}$, and ${\tau }_{B\to D}$. The calculated values were obtained by multiplying the expected rate constant ${k}_{C\to D}$ and ${k}_{B\to D}$ from visual DSD by the Re concentration. This was followed by comparing the forced and expected (Visual DSD) kinetic traces (Fig. SI 14). The comparison showed that the forced and predicted kinetic traces diverged significantly, indicating that the DLRN analysis was correct. Therefore, the discrepancy between the expected and predicted tau values is not associated with the neural network’s performance but with possible non-first-order kinetic mechanisms.

This discrepancy indicates that the B → D and C → D pathways have more complex dynamics and that multiplying the expected rate constants by the Re concentration is not sufficient to calculate the correct time constants. To extrapolate the correct rate constant, DLRN was used to analyze four data sets with different Re concentrations. After DLRN analysis, the inverse of ${\tau }_{C\to D}$ obtained from DLRN was plotted versus Re concentration and then the data fitted using a linear regression (Fig. SI 15). Using this method, second-order reaction pathways can be tracked and the expected rate constant *k*_0_ (intercept from the linear fitting) can be calculated. The intercept value obtained from the linear regression is 4.83 × 10^−5^, which differs from the expected value, *k*_CD_ = 4.62 × 10^−5^, by approximately 4.5%.

**Fig. 6:**
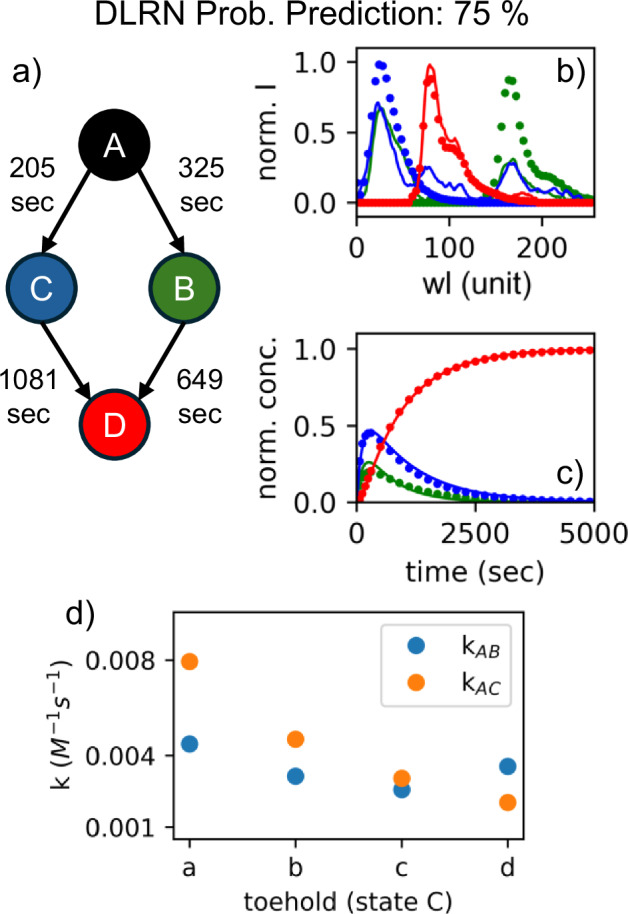
Full DSD kinetic reaction analysis. **a** DLRN model prediction. **b** Expected (filled circles) and DLRN-predicted (continue line) spectra for the full DSD dynamics. Colors correspond to the kinetic model in **a**. **c** Expected (filled circles) and DLRN-predicted (continuous lines) kinetic traces for DSD dynamics. Colors correspond to the kinetic model in **a**. **d** Rate constants *k*_AB_ (blue) and *k*_AC_ (orange) obtained from DLRN analysis of four variant Input strands by changing the toehold of the DNA strand.

The DLRN was also used to analyze the case in which both Su1 and Su2 are present and can interact with the input strand, thus generating a branching mechanism for the formation of the final state D. Figure 6 shows the results obtained from DLRN analysis using Inp = 10 nM, Su1 = 12 nM, Su2 = 8 nM, and Re = 30 nM. The neural network was able to establish the correct network architecture with a probability of 75% (Fig. 6a) and correctly reproduced the kinetics of the dynamics (Fig. 6c), indicating that the predicted tau values represent each decay pathway. However, the amplitude was not well disentangled from the analysis in which the spectra of the states C and B mixed with each other. Despite differences in expected amplitude, residuals from DLRN analysis were good (Fig. SI 16a), indicating a correct analysis of the dynamics. This behavior can be attributed to the identical rising-decaying kinetics of the states C and B (Fig. SI 16b), which makes them extremely difficult to disentangle. We also tried to analyze the data by classical fitting using a branching mechanism. For the fit, a branch coefficient between 0.01 and 1 for state A, and positive amplitudes were imposed. Surprisingly, the DSD CRN was better analyzed by DLRN than by classical fitting (Fig. 6 and Fig. SI 17). Kinetic traces and time constants were better resolved by DLRN, whereas classically fitted traces deviated significantly from those expected. Conversely, classical fitting extrapolated cleaner and separate amplitudes. However, the spectrum of B differed greatly from the expected position, which was not the case with DLRN.

We analyzed the DSD dynamics, which had a series of different Inp strands with varying toehold (varied domain 1 of the Inp DNA strand, blue sequence Table SI 4). Because the corresponding toehold mediates the reaction rate of each DSD step, this modification affects the formation of state C. The longer/stronger the toehold binding, the faster the migration of the whole DNA-branch from Inp to Su1. We designed four different DNA toeholds that decelerate the kinetic pathway A → C, and extrapolated the rate constants *k*_AC_ and *k*_AB_ using DLRN. The concentrations of Inp, Su1, Su2, and Re were kept constant at 10 nM, 10 nM, 10 nM, and 30 nM, respectively, and the results are shown in Fig. 6e. DLRN was able to track the variation of *k*_AC_ for the toeholds used, predict the correct branching mechanism, and follow the expected kinetic traces (see also Fig. SI 18). The kinetic pathway from state A depends on *k*_AB_ and *k*_AC_ values, and it was possible, especially from the kinetics traces, to observe preferences. With toehold a, population of state C is preferred, whereas for toehold d, decay into B is preferred.

### G-User-DLRN: a graphic user interface for DLRN analysis

To increase the method’s usability by obtaining a DLRN analysis in a few concise steps, a Python-based graphical user interface (GUI) was developed (G-User-DLRN). After running the file “DLRN_GUI”, a graphical interface starts, and several options are available for the user to select to begin the analysis. The first option allows the user to use the pre-trained DLRN model for spectral (PL and TA) or agarose gel analysis. The button “load custom scale” loads the .txt file containing a vector of time points used for the measurements. This allows the rescaling of data to match the timescale used to train the DLRN. The loaded data is shown in an additional window after selection via the “load data” button. The GUI also allows the Top 1 and Top 3 analysis outputs to be chosen. After these selections are made, clicking the “data analysis” button generates a number of windows depending on whether Top 1 or Top 3 was chosen; each window shows the complete analysis for the most probable (Top 1) or the three most probable solutions (Top 3). Each window shows a kinetic model representation with the DLRN probability, an indication of how confident the DLRN is regarding a particular outcome.

Additionally, tau, amplitude, and population profiles obtained by DLRN analysis are shown in the window for each prediction. Moreover, residuals normalized to the maximum value of the dataset are presented in the window to understand how representative the predictions are of the data. The analysis also provides a table showing the time constant value predicted by DLRN and the model prediction in one-hot encoding representation. At the end, G-User-DLRN has a button called “test DLRN”, which performs the analysis on test data, but with the difference that expected values for model, tau and amplitudes are shown in the result’s windows. This option can be used to check the performance of DLRN on ground truth data sets. Additional information, including a primary pipeline and images of G-User-DLRN can be found in the Supplementary note 7.

### Analysis of experimental data sets using G-User-DLRN interface

**Fig. 7:**
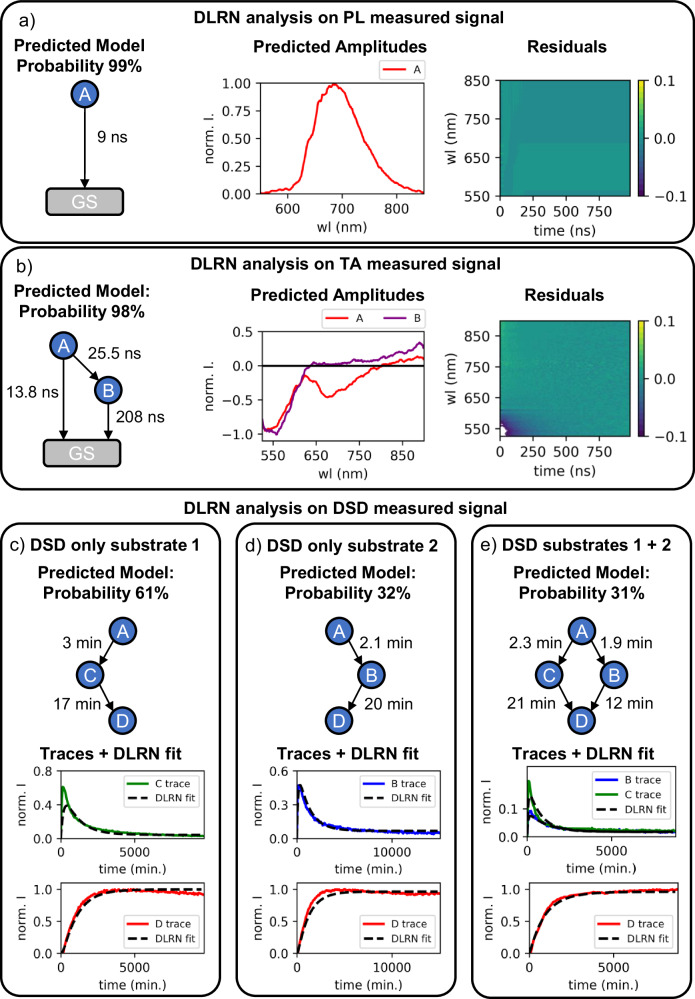
Experimental data analysis using DLRN. **a** DLRN analysis on time-resolved PL of negatively-charged Nitrogen-vacancy (NV-) centers in diamond. **b** DLRN analysis on TA spectroscopy of NV centers in diamond. **c**–**e** DLRN analysis on DSD systems where only Su1 (**c**), only su2 (**d**) or both Su1 + Su2 are present. Each analysis shows the predicted model with tau values for each decay path. Additionally, predicted activate state amplitude, residuals of trace + DLRN fit comparison are shown.

We used the DLRN interface to analyze experimental measured data sets from three samples with different dynamic complexities (Fig. 7). To use G-User-DLRN interface, the data sets have to be preprocess following a specific pipeline (see “Preprocessing pipeline for experimental data analysis using DLRN” in methods), which include baseline correction, removing the coherent artifact and time zero correction. Initially, we analyze the negatively-charged Nitrogen-vacancy (NV^-^) centers^60–62^ emission measured with time-resolved photoluminescence spectroscopy (Fig. 7a). This system has a simple decay dynamic A → GS between 9.5 and 10 ns, which is a nice starting experimental example to test the neural network. DLRN predicts a single decay dynamic to the ground state with a probability of 99% (Fig. 7a, predicted model) that has a decay time constant of 9 ns, which is in line with the expected result^60^. DLRN also predicts a broad band spectrum from 600 nm to 850 nm (Fig. 7a, predicted amplitude), which combined with the kinetic model shows good residuals (Fig. 7a, Residuals). This suggests that the amplitude was correctly predicted by the neural network. The second sample analyzed by DLRN was NV centers in diamond measured with TA spectroscopy (Fig. 7b)^63^. In this case, the system shows a more complex dynamics, where the first excited state can populate through a branching mechanism both the ground and a second excited state, while the second excited state goes to the GS^63^. In simple terms, the systems dynamic is a combination of A → B → GS and A → GS. Also in this case, DLRN predicts correctly the kinetic model with a probability of 98% (Fig. 7b, predicted model), showing *τ* values that are in line with previous reported work^63^. In fact, we can observe that A decays with a total value of *τ*_*A*_ = 9 ns, while B with *τ*_*B*_ = 208 ns, which are in line with the respective expected values of 9.5 µs and 200 ns. DLRN can also extract two exited state spectra that show different features (Fig. 7b amplitude prediction). The Good performances of DLRN analysis are also supported by the good residuals, which only show a negative contribution in the initial dynamics of the ground state bleach area (Fig. 7b, Residuals). The last sample analyzed by DLRN are three DSD samples for one of the toeholds presented before (toehold d) in the case when, Su1, Su2 or Su1 + Su2 are present (Fig. 7c–e). In detail, we expected the same kinetic reaction model (with A as a dark state) that are shown in Fig. 5d, Fig. 5e and Fig. 6a for Su1, Su2 and Su1 + Su2 respectively. In these three cases, we were able to measure sparse kinetic traces measuring emission spectra over time. For all of them, DLRN was able to extract the expected kinetic reaction model from the Top 2 of the most probable predicted models (Fig. 7c–e, predicted model). Regarding the tau values for Su1 and Su2 only, we could approximate estimates the expected tau values supposing a pseudo-first reaction. In the case of Su1 only (Fig. 7c), approximate tau values were ${\tau }_{A\to C}$ = 1.2 min and ${\tau }_{C\to D}$=16min, while DLRN analysis shows comparable tau values of ${\tau }_{A\to C}$ = 3 min and ${\tau }_{C\to D}$ = 17 min. In the other case of Su2 (Fig. 7d), the extracted tau values were ${\tau }_{A\to B}$ = 1.8 min and ${\tau }_{C\to D}$ = 18 min, which are similar to the DLRN predicted values of ${\tau }_{A\to B}$ = 2.1 min and ${\tau }_{B\to D}$ = 20 min. For the more complex system where both Su1 and Su2 are present (Fig. 7e), it was not possible to give approximate tau values due to the branching mechanism. However, the comparison between the kinetic traces and DLRN fit shows that the extracted tau value, amplitude and kinetic model from DLRN can properly approximate the kinetic reaction of the system (Fig. 7e, Traces + DLRN fit), giving tau values of ${\tau }_{A\to B}$ = 1.9 min, ${\tau }_{A\to C}$ = 2.3 min, ${\tau }_{B\to D}$ = 12 min and ${\tau }_{C\to D}$ = 21 min for the Su1 + Su2 system. Additionally, DLRN predicted correctly the kinetic reaction model and the differences between fit and kinetic traces could be linked to the few and sparse kinetic traces measured, which DLRN was not trained for. Nevertheless, DLRN has managed to analyze, in a good approximation range, the DSD data sets.

## Discussion

We have introduced DLRN, a deep learning framework for the GTA of 2D time-resolved data sets. The standard approach of fitting kinetics using GTA is almost compulsory for the analysis of dynamic systems because it can quantitatively extrapolate kinetic mechanisms from time-resolved data^5,15–18^. However, the path toward achieving results can be long and complex, requiring a lot of time and an interwoven approach of mathematical modeling, assumptions, and experimental data. Furthermore, even with a good approach that guides one through the analysis, a family of models might show the same residual performance and reasonable results, making it difficult to distinguish from the analysis alone and requiring further work to identify the “best” solution. Indeed, this was the case during the fitting algorithms test (Fig. 2c, d and Fig. SI 8), where target fitting of those algorithms gave suitable residual and reasonable model parameters, but different from expected ones. Moreover, the kinetic model was already known and used for the analysis, which drastically reduces the workload for a classical analysis by excluding all the other possible models that can fit the data. Conversely, DLRN alone could extrapolate the correct kinetic model with a confidence of 98.9% (Fig. 2b). Moreover, it could correctly conclude (within an error of about 5%) both the time constants and the amplitude shape (Fig. 2b). Classical fitting and KiMoPack were only superior in terms of residual scores, which were higher with DLRN, although the two were somewhat comparable in this aspect. This arises because the DLRN neural network yields a fixed solution with a margin of error. Indeed, the analysis performed on a test batch showed excellent performance (Table SI 1) but with some flexibility. One of the critical roles of flexibility lies in the Top 1 and Top 3 solutions. The performance of DLRN shows that the neural network can become confused and not clearly distinguish one solution from another. However, it gives a maximum of three possible models (each has its amplitude and time constants), and the probability that the real solution is one of these is greater than 98%. At this point, the probability confidence (y value in Fig. 1b) can be combined with the residuals between the DLRN results and data to obtain the most probable solution that best reflects the system. The speed of DLRN is remarkable, it can extrapolate a complete analysis in seconds.

An exciting aspect of DLRN is that it can be used to analyze more than one type of time-resolved data. DLRN was used to analyze both time-resolved agarose gel electropherograms and transient absorption (Fig. 4). Compared to the emission spectral analysis, DLRN performance with gels was lower (compare Tables SI 1 and SI 2), but still good (Figs. SI 11–13), while the TA performances were higher (compare Table SI1 and SI3). In The case of DLRN gel predictions, the most noticeable differences were observed for the predictions of time constants and amplitudes, while model predictions differed by less than 2% in both Top 1 and Top 3 values compared to the spectral analysis. Differences in tau and amplitude values can be expected due to the nature of the agarose gel, which is made up of cells, thus reducing the number of actual points carrying dynamics information. On the Contrary, DLRN TA predictions shows a higher Top 1 model prediction (5% difference), while Top 3, time constants and amplitude predictions are similar to DLRN PL predictions.

Another crucial point was to evaluate DLRN for multi-timescale analysis to determine whether the neural network is capable of analyzing complex dynamics that are split into different time scales (Fig. 3). The neural network was used to analyze a signal that evolves into three timescales, from the nanosecond to the millisecond, which involves more species than those used during training. The signal was decomposed into three data sets, each with only one timescale, and subsequently analyzed by DLRN. DLRN was able to extrapolate the correct kinetic model and its parameters, making it possible to reconstruct the complex expected dynamics. This shows that DLRN can be used to analyze data sets on more than one timescale and for kinetic reaction mechanism with more than four active states. Indeed, DLRN architecture is thought to extrapolate four species for each timescale. This means that if more than four species are involved in the dynamics but split over several timescales, DLRN can theoretically extrapolate all of them by analyzing each time window individually.

DLRN was used to analyze DSD CRNs in different conditions to emulate its utility for complex systems under certain conditions. The expense of DNA limits the number and type of experiments from which one can collect data for classical fitting. In addition, classical methods cannot predict certain effects (e.g., enzymatic steps or pH-dependence). Although DSD reactions are usually second-order, for both linear (Fig. 5) and branching (Fig. 6) mechanisms, DLRN was able to correctly reproduce the DSD kinetics (pseudo-first-order-reaction limited), predicting kinetic model, time constants, and amplitudes of the systems. DLRN was also compared to the classical fit for the branching mechanism (the most complex one), and surprisingly, the neural network could better reproduce the system’s dynamics (Fig. 6 and Fig. SI 17). Moreover, using DLRN, we were able to extrapolate the correct rate constant *k*_CD_ within an error of 4.5% (Fig. SI 15) and the kinetics for four different DNA toeholds for the full dynamics with Su1 and Su2 both present (Fig. 6e). Together, these results demonstrate the efficiency of DLRN in kinetic analysis and its versality for systems of varying complexity.

DLRN was also used to analyze NV and DSD experimental datasets (Fig. 7). Initially, we analyze the NV^-^ emission dynamics, which show a simple decay pathways of the excited state to the GS (Fig. 7a)^60^. DLRN was able to correctly predict the model with comparable time constant values (5% difference between expected and predicted taus). The second system analyzed by DLRN was again a NV center, but in color centers in diamond^63^, which shows a branching kinetic mechanism (Fig. 7b). Also in this case, DLRN was able to accurately reproduce the dynamics. DLRN also analyzed experimental DSD sparse kinetic traces (Fig. 7c–e). In detail, we analyzed the kinetic reaction when Su1, Su2 or Su1 + Su2 were present in the measured sample. In all the three cases, DLRN was able to predict the correct kinetic model from the Top2 most probable models (see model predictions, Fig. 7c–e). Comparing DLRN fit and kinetic traces, DLRN seems to reproduce the dynamics of the systems, but with more errors. However, it is important to note that although DSD systems trend toward a second-order reaction and sparse kinetic traces were measured, DLRN was able to extract, within a margin of error, the correct kinetics.

In this work we show that DLRN can be used in several scenarios, including real data measurements. However, the neural network has still some limitations for kinetics analysis, which opens the way for possible future improvements of DLRN. One of these aspects is the limitation of DLRN to the analysis of first or pseudo first kinetic reactions. However, including higher order reactions may not be an easy task due to the fact that the number of kinetic models scales a lot. For example, including only one possible second order path on top of the 102 models that DLRN can analyze, the total number of kinetic models scales to 500+. This could reduce the neural network performance, which could require severe architecture adjustments to obtain the best analysis performances. On parallel, the time constants block should be thought differently because first order rate constants *k*_first_ depends only om the time (s^−1^), while second order *k*_second_ depends also on the concentration (s^−1^ M^−1^). Another important aspect to consider is the data training pool, with could also scale a lot due to the higher number of kinetic models. These are a few aspects to consider for deep learning and high order reactions. However, the results already obtained with DLRN may be a good basis for expanding the neural network on a higher order level for the analysis of nonlinear systems, such as second order reactions. This future work could bring an alternative deep learning algorithm for higher order reaction analysis.

## Methods

### Neural network architecture

The core methodological component proposed here is a combination of an architecture based on Inception-Resnet^56^, which is composed of four blocks (stem, ConV. A, ConV. B, and ConV. C) and three personalized output blocks (model, time, and spectrum). This approach created a deep learning reaction network (DLRN) for analyzing 2D time-resolved data sets, as schematically visualized in Fig. SI 1. A more detailed representation of the architecture is shown in Fig. SI 1. As a general overview, each Inception block comprises several convolutional and pooling layers in parallel, where kernel size, filters, and resize depends on the specific block. Each block is repeated in the network architecture several times: once for stem, five for block A, 10 for block B, and five for block C.

Additionally, two types of reduction modules are introduced after the total repetitions for both A and B blocks. In the case of the output blocks, the architecture changes depending on the desired output (Fig. SI 2). The model block determines the kinetic model that best represents the data over 102 possible kinetic models. It comprises an averaging pool layer, a dropout layer, and a dense layer with a Softmax activation function (Fig. SI 2a). The categorical cross-entropy loss function is used to optimize the model prediction. The time block was designed to extrapolate the kinetic time constants associated with the kinetic pathways of the obtained model. This block has two inputs: the output from the last block C, and the concentration matrix linked to the specific kinetic model in the analysis. The two inputs are processed in parallel using several dense layers and residual convolutional blocks to obtain the number and the value of the time constants involved in the dynamics using a regression problem (Fig. SI 2b). The logarithm of the hyperbolic cosine was used as a loss function for the time block. The amplitude block extrapolates the number and the shape of the species involved in the dynamics, which are linked to the amplitudes of each decay component that contributes to the signal. Similar to the time block, the amplitude block also has two inputs: the output from the last block C and the concentration matrix (see also Fig. SI 2C). The two inputs are initially analyzed in parallel and then merged and sent through several separable convolutional and convolutional layers. As with the time block, the linear activation function was used for the last layer, and the mean squared error was applied as a loss function. The neural network was trained using about 2 million synthetic images each with a size of 256 × 256 × 1. The Adam optimizer was used for the training with a starting learning rate of 0.0001.

### Generation of synthetic data for training and evaluation

The DLRN deep neural network was created with the aim of analyzing, as best as possible, one time scale at a time under selected conditions. To obtain an optimal and comprehensive data set to achieve this task, and due to the limited experimental data available, we used synthetic data to train and evaluate the neural network. For this purpose, a kinetic model with five electronic states (four excited states and one ground state) was randomly created to produce the respective synthetic data sets. However, some constraints were applied to the generation of the model, such that only physically feasible models could be generated (a total of 102). The assumptions used to reduce the complexity of the prediction task were (1) there is only one initial populated state, (2) a forward reaction without cycling; (3) each active state can only populate a maximum of two other states. Restrictions 1 and 2 are physically motivated since perturbed states are not in equilibrium, and only one molecule is usually initially triggered. Assumption 3 is made for practical reasons to reduce the total number of possible models. Moreover, it is decidedly challenging to distinguish branching between three or more states in kinetic reactions, so the third assumption is a reasonable restriction.

The data sets for training and evaluation were generated by simulating time-resolved data **X**(t, λ) for each physical kinetic model. Two components are necessary to create this data: the active spectra matrix **S**(λ) of the electronic state and the concentration trace matrix **C**(t) of the selected kinetic model. The product **C**(t)*S(λ) generates the 2D signal **X**(t, λ), which is the sum of all the spectra at each time delay. Spectra of each state **S**_**i**_(λ) were generated by summing a random number of one to ten Gaussian functions with random peak position (between 0 and 256 every 2 indexes), width (between 3 and 25), and intensity (between 0.2 and 1). Notably, at least one peak of each spectrum has a minimum intensity of 0.4. This allows generation of sequential spectra with complex structures from the initial to the last populated state. In the case of transient absorption TA 2D signal, a negative component was added to each spectra to simulate the difference in absorption $\Delta {A}_{{signal}}={A}_{{exc}.}-{A}_{{GS}}$ between excited and ground states. The ${A}_{{GS}}$ signal was generated with the thought of simulating the sum of the ground state bleaching and the stimulated emission bands and it was created as the same as each **S**_**i**_(λ). The concentration matrix **C**(t) was created by converting the kinetic matrix associated with the randomly selected kinetic model into differential equations using a fifth-order Runge–Kutta method. The time constants for active pathways were randomly generated in sequence (from ${\tau }_{1}$ up to ${\tau }_{n}=7$, depending on the number of active species from the kinetic model). For each ${\tau }_{n}$, a ${\tau }_{\min }$ and ${\tau }_{\max }$ values were selected between 1 and 605 generating a specific subset for ${\tau }_{n}$ (see Supplementary note 3 and Fig. SI 9 for more info). Next, ${\tau }_{n}$ was randomly selected between ${\tau }_{\min }$ and ${\tau }_{\max }.$ As mentioned above, DLRN was created to analyze one time window at a time, which makes the larger value of ${\tau }_{n}$ = 605 a good limitation as the decay should be enough represented in the data. The time points were interpolated by combining a linear growth scale with an exponential growth scale to obtain a total of 256 time points. To make the 2D signal more realistic and to avoid overfitting (including dropout layers, see fig. SI 1-2), a random gaussian noise with center to zero and a possible sigma value of 0.005, 0.01 or 0.02 was added to each training data. As mentioned before, about 2 M of datasets (~20k 2D data for each kinetic model) were created for the fitting due to the complexity of spectra and time constants variability that could be created for each kinetic model.

### Generation of DSD data for testing the DLRN

The DNA sequences of the domains were designed intuitively, and their crosstalk was avoided by checking their interactions using NUPACK^64^. The stem sequences are gray, and the toeholds are colored (Table SI 3). Table SI 3 shows the exact DNA sequences with the fluorophores and quenchers. The rate coefficients of binding and unbinding of complementary DNA sequences were derived as shown in Supplementary note 8, and the values are summarized in Table SI 4.

### Generation of experimental data to test DLRN

Nitrogen-vacancy (NV) centers and DNA strand displacement (DSD) samples were measured using photoluminescent (PL) and transient absorption (TA) experimental techniques. Starting with the NV centers, we used a type 1b diamond substrate that was synthesized through a high-pressure high-temperature process, containing a substitutional nitrogen concentration of approximately 100 ppm. Vacancies were created in the substrate by electron irradiation with an energy of 1 MeV, and subsequent high-temperature annealing was performed to achieve an NV concentration of around 1.5 ppm. The dimensions of the sample are 3 × 3 × 0.4 mm^3^. For the TA measurements, the same setup as the one published before was used^60,63^. In detail, a pulse centered at 515 nm is used to excite the sample, while a broad band CW white-light source was used to track the ΔA signal over time. In the case of PL measurements on NV centers sample, same setup was used, but the WL was switched off to measure the emission signal.

In the case of the DSD sample, the DNA strands were purchased from Biomers GmbH (see Table SI 4 for sequences) and were used without further purification. First, stock solutions of single stranded DNA (ssDNA, 100 μM) were prepared in TEA buffer (40 mM Tris base (Sigma), 1 mM Ethylenediaminetetraacetic acid disodium salt dihydrate (Roth), pH = 8 set by glacial acetic acid (VWR)). Then stock solutions of double stranded DNA (dsDNA, 20 μM) were prepared from equimolar mixtures of ssDNA stock solutions in TEAM buffer (TEA buffer with 12.5 mM magnesium acetate tetrahydrate (Roth)) by annealing the mixture at 95 °C for 5 min and cooling down to 20 °C with the rate of 1 °C/min. DNA concentrations were determined using a DeNovix-S-06873 spectrophotometer. The stock solutions (500 nM) were made using MilliQ water and were stored in the freezer. The reaction mixtures were prepared in a Corning® 384-well black polystyrene plate with non-binding surface in a total volume of 20 uL. Precise volumes of the reagents were pipetted and mixed using an automated microvolume liquid handler (Dispendix I.DOT). After mixing all components but Input, 10 uL hexadecane was added on top for isolation and to avoid evaporation. The plate was incubated at 37 °C for 10 min in a TECAN (SPARK control v3.1) microplate plate reader. The reaction was started by adding a few uL 500 nM Input stock solution on top of the hexadecane, and a short intense shaking in the plate reader triggered the mixing of the Input droplet with the rest of the aqueous reaction mixture. The fluorescence recording by the plate reader started instantaneously. Data was collected in every 60 sec with the following excitation and emission wavelengths: 665 nm and 705 nm for State B (Cy5.5), 440 nm and 485 nm for State C (Atto425), and 525 nm and 575 nm for State D (Cy3). Fluorescent intensities were normalized to baseline intensity recorded for 10 min before starting the reaction.

### Preprocessing pipeline for experimental data analysis using DLRN

To analyze the experimental data, it is necessary to preprocess the signal to obtain a correct performance from DLRN. The data Initially has to be baseline corrected and time zero corrected. Additionally, the coherent artifact has to be removed from the signal. In this work, a Gaussian model was used for removing the coherent artifacts from the signal, which gives you t_0_ (center of the Gaussian model) and σ (standard deviation of the gaussian model. After that, the time scale of the data was adjusted time using the following formula:

1 $${TimeScal}{e}_{{new}}={TimeScal}{e}_{{old}}-({t}_{0}+\sigma )$$

In this way, we ensure that any residual effect of the coherent artifact is removed. We also observed that sometimes DLRN performances can be slightly improved using $({t}_{0}+2* \sigma )$ for the generation of the new time scale. After these steps, the data can be analyzed using the user graphic interface of DLRN, which loads and interpolates the 2D data set to create an exact image of 256 × 256 pixels for the analysis. The preprocessed 2D data has to be saved in a .txt or .npz file before loading.

### Area metric for regression accuracy

To determine the accuracy and power of the regression analysis, we introduced the measure of area metric *A*_M_. This measure is calculated from the ratio between the residuals and the areas of the expected values (Eq. 1). Because Δx of the data is constant, the ratio can simply be expressed as the sum of the residuals (**Res**) divided by the sum of the expected values (**ExpVal**).

2 $${A}_{M}=1-\frac{{\sum}_{i}\left|{{\boldsymbol{Re}}}{{{\boldsymbol{s}}}}_{i}\right|}{{\sum}_{{{\boldsymbol{i}}}}{{\boldsymbol{ExpVa}}}{{{\boldsymbol{l}}}}_{i}}$$

*A*_M_ is a value between 0 and 1, where 1 indicates that the two areas overlap entirely. By setting a lower bound for good area overlap, it is possible to determine how many predictions of the entire batch can be considered a positive result. This reduces the problem to a binary one, where accuracy can be calculated by dividing the number of positive predictions *N*_p_ by the total number of samples *N*_tot_ (Eq. 2):

3 $${Accuracy}=\frac{{N}_{p}}{{N}_{{tot}}}$$

Throughout this study, we set the lower limit on an excellent area overlap at 0.8 and 0.9 for the predictions of time constants and 0.8 for amplitude predictions (the choice of these limits is explained in the Supplementary note 4.

### Re-training pipeline for DLRN

DLRN code is publicly available (https://github.com/mem3nto0/G-User-DLRN) and it could be used for re-training the neural network. For doing this, some expediency must be followed. The following steps are linked to data Generation (see above) and to the aim of the new training:

1. Input image: New input image size and DLRN architecture input size has to be matched by changing the input layer of DLRN.
2. Model prediction: the output dimension of the softmax dense layer has to be changed according to the total number of models that the user wants to train. It is important that during data generation the models are labeled differently in position in the one-hot encoding representation.
3. Time constants prediction: The output dimension layer should be changed according to the higher number of possible decay pathways that a model can generate. For time constants predictions, the DLRN block needs a second input, which is the reference matrix (See Eq. SI 3 and 4). Matrix dimension has to be changed depending on the total number of possible active states.
4. Amplitude prediction: The output dimension layer should be changed according to the higher number of possible active state or excited state. For Amplitude predictions, the DLRN block needs a second input, which is the reference matrix (See Eq. SI 3 and 4). Matrix dimension has to be changed depending on the total number of possible active states.

It is important to underline that DLRN input and outputs are strictly linked to data generation, which we suggest being careful during DLRN re-training to avoid mistakes.

## Supplementary information

Supplementary Material

## Data Availability

All data used to evaluate the conclusions in the paper are presented in the result and discussion of this manuscript and in the supplementary information. Examples of the data used in this paper have been deposited in the git-hub page under the section https://github.com/mem3nto0/G-User-DLRN “Test_data_APP”.

## Code availability

The code is available at https://github.com/mem3nto0/G-User-DLRN.
