# Supplementary material for: Deep Learning Reaction Framework (DLRN) for kinetic modeling of time-resolved data
Source: Commun Chem. 2025 May 15;8:153. doi: 10.1038/s42004-025-01541-y (PMC12081648; doi:10.1038/s42004-025-01541-y)
Supplement: Supplementary file 1 — Supplementary Material [file 42004_2025_1541_MOESM1_ESM.pdf]

Deep Learning Reaction Framework (DLRN) for kinetic modelling of time-resolved data

Supplementary Information

Supplementary note 1: Deep learning reaction network (DLRN) architecture

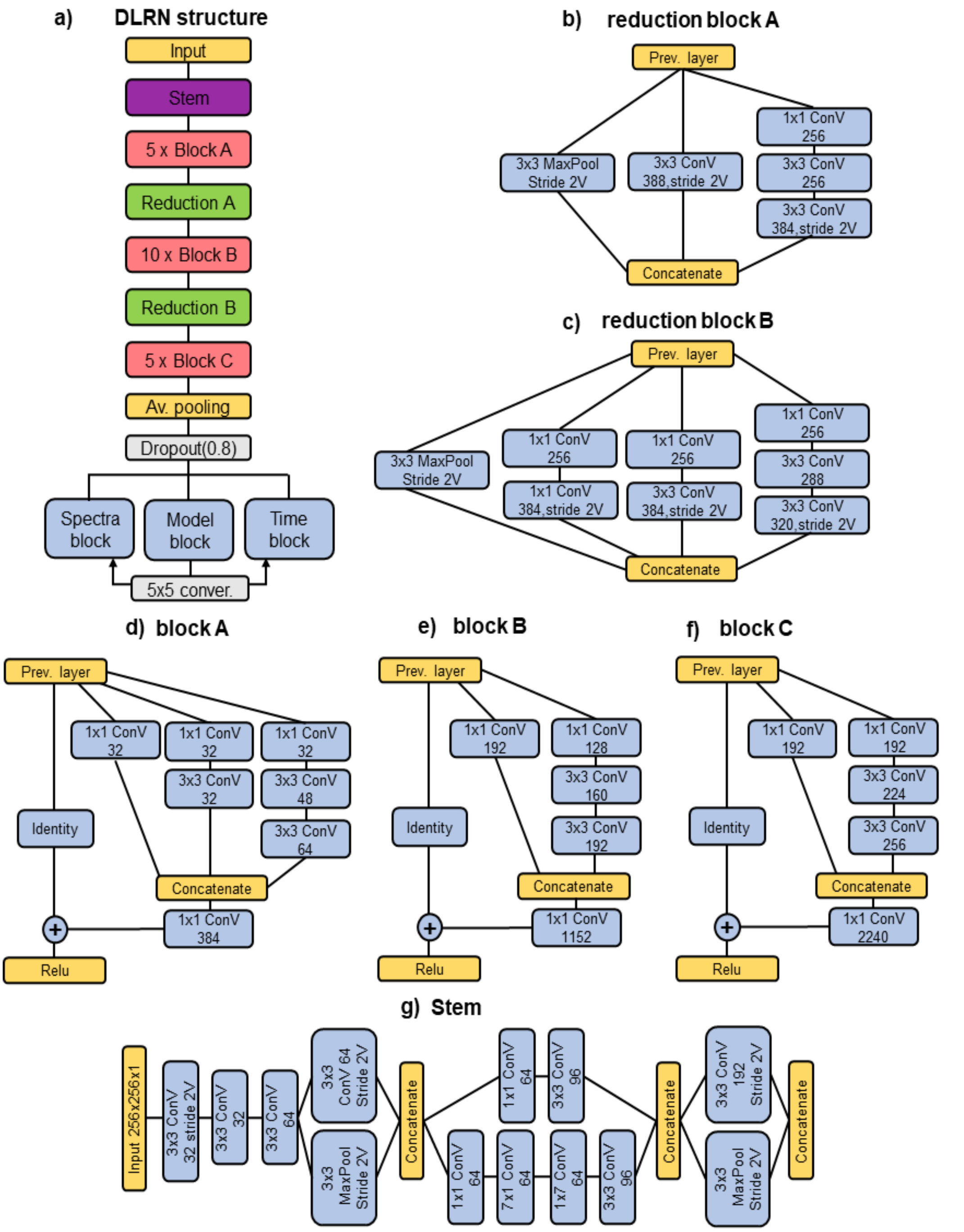

Fig. SI 1. a, Full architecture of the DLRN neural network. **b,** Reduction block after A blocks. **c,** Reduction block after B blocks. **d,** Residual block A. **e,** Residual block B. **f,** Residual block C. **g,** Stem block.

Fig. SI 1 shows the entire architecture of the DLRN, which has about 100M of parameters. Input data—images with dimensions 256×256×1; time and wavelength in the x and y axes, respectively—are sent through the neural network to extrapolate the model, time constants, and spectra from the 2D data. Model, time, and spectra blocks are shown in Fig. SI 2.

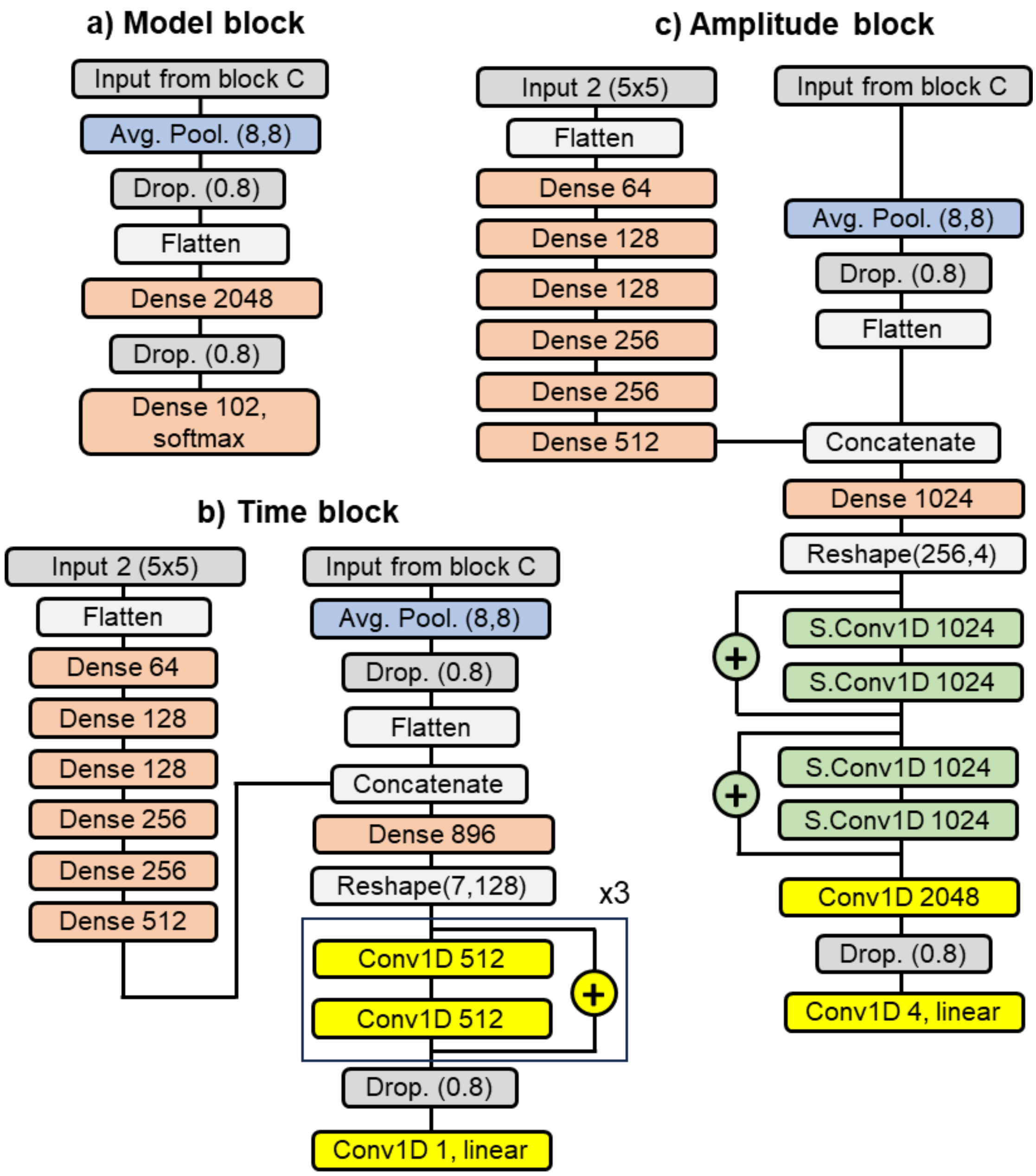

Fig. SI 2. a, Model block. Input from the last block C is sent through averaging and a dense layer, which uses Softmax activation. **b,** Time block. The model block provides one of the inputs after conversion in a concentration matrix reference. In parallel, block C's output is a second input. The two inputs are processed and then concatenated

and sent through three residual convolutional blocks. Linear activation is used for the last convolutional block after dropout. **b,** Amplitude block. As for the time block, the two inputs are converted outputs from the model block and block C. To obtain the amplitudes, the two inputs are elaborated using dense, separable convolution 1D (S.Conv1D), and convolution (Conv1D) layers. A linear activation function is used in the last layer.

Supplementary note 2: Generation of Training input and output datasets

We created a kinetic model to generate a synthetic 2D data set as an input for the training. Kinetic models can be expressed by a set of differential equations. For example, for the linear model A→B→GS (where GS is the ground state), we are considering only four possible active states, so the system of differential equations can be written in the following matrix form:

$$\begin{bmatrix} dA/dt \\ dB/dt \\ dC/dt \\ dD/dt \end{bmatrix} = \begin{bmatrix} -k_1 & 0 & 0 & 0 \\ k_1 & -k_2 & 0 & 0 \\ 0 & 0 & 0 & 0 \\ 0 & 0 & 0 & 0 \end{bmatrix} \begin{bmatrix} A \\ B \\ C \\ D \end{bmatrix} \quad \text{(Eq. SI 1)}$$

A, B, C, and D are the active states and k_1 and k_2 are the kinetic rate constants (which are the inverse of the time constants involved in the mechanism). The system of differential equations can be resolved by introducing the initial conditions, typically [A(t_0),0,0,0], and assuming that initially only the active state A is populated. The solution gives us the concentration trace matrix **C**(t), which can be written like a system of equations:

$$\begin{cases} A(t) = A_0 e^{-k_1 t} \\ B(t) = A_0 \frac{k_1 - k_2}{k_1 k_2} (e^{-k_2 t} - e^{-k_1 t}) \\ C(t) = 0 \\ D(t) = 0 \end{cases} \quad \text{(Eq. SI 2)}$$

To complete the generation of the concentration trace matrix **C**(t), it is necessary to generate the values of the rate constants to be substituted within the equations. To this end, a set of about 210 time constants between 1 and 605 was generated with a nonconstant step (step = 1 between 1 and 20; step = 2 between 20 and 250; step = 5 between 250 and 600). Depending on the number of active states that contribute to generating the signal, variable subsets were created for each state. In our example of the linear mechanism above (Eq. SI 2), the only states contributing to the signal are A(t) and B(t), meaning that two subsets have emerged. This means that in the case of

one active state, only one subset is generated (which in this case is the entire set); in the case of two active states, two subsets are created, and so on.

Fig. SI 3 shows how subsets are made variably. Starting with the creation of the subset for the state A(t), a random index is chosen between minimum and maximum values. After the selection, the subset for A(t) is made, and a new initial set is generated for the creation of the subsets for B(t), and this process is repeated for all the active states. The decay time constants were then selected randomly from the appropriate subset for each active state. The reasons we decided to generate the tau values in this way are two: 1) we wanted state n+1 decay more slowly than state n; 2) we wanted to generate as many cases as possible that satisfy condition 1. In the case of a parallel mechanism for an active state, the two decay time constants were also selected from the corresponding subsets, but with the condition that the quantum yield

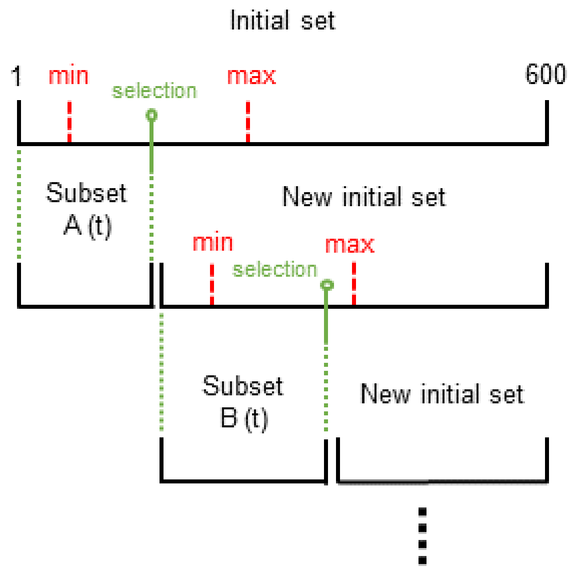

for a pathway is between 30% and 70%.

Fig. SI 3. Scheme for generating the dynamics subset for the active electronic state.

As mentioned in the main text (see Methods, Generation of synthetic data for training and evaluation), to generate the signal **X**(t,λ), we also need the amplitude matrix **S**(λ). For each active state, we needed to generate the corresponding spectrum (the amplitudes of the active state). To do so, each spectrum was generated with a sum of a random number of Gaussians between one and eight, where each Gaussian can have an amplitude between 0.2 and 1 and a width between 5 and 50. Notably, at

least one Gaussian has a minimum amplitude of 0.4. After that, all spectra were collected to generate the amplitude matrix **S**(λ), where the first row is the amplitude vector of the active state A, the second row for the active state B, and so on.

The output training data were generated differently depending on the output block (model, time, and amplitude; Fig. SI 2). Starting with the model block, each kinetic model can be represented by a reference matrix $\mathbf{M_r}(k)$, which is linked to the *k* matrix part shown in Eq. SI 1. In detail, using the same k matrix as in Eq. SI 1, it is possible to write the $\mathbf{M_r}$(k) for this kinetic model as follows:

$$\boldsymbol{M_r}(k) = \begin{bmatrix} -1 & 0 & 0 & 0 \\ 1 & -1 & 0 & 0 \\ 0 & 0 & 0 & 0 \\ 0 & 0 & 0 & 0 \end{bmatrix} \approx \begin{bmatrix} -k_1 & 0 & 0 & 0 \\ k_1 & -k_2 & 0 & 0 \\ 0 & 0 & 0 & 0 \\ 0 & 0 & 0 & 0 \end{bmatrix} \qquad \text{(Eq. SI 3)}$$

Here, the rate constants are replaced by the value 1, which indicates whether a state decays or is formed during the process. Another case to consider is that of a branching mechanism. Eq. SI 4 shows an example of how $\mathbf{M_r}(k)$ can be written in the case of a parallel mechanism:

$$\boldsymbol{M_r}(k) = \begin{bmatrix} -2 & 0 & 0 & 0 \\ 1 & -1 & 0 & 0 \\ 1 & 0 & 0 & 0 \\ 0 & 0 & 0 & 0 \end{bmatrix} \approx \begin{bmatrix} -(k_1+k_2) & 0 & 0 & 0 \\ k_1 & -k_3 & 0 & 0 \\ k_2 & 0 & 0 & 0 \\ 0 & 0 & 0 & 0 \end{bmatrix} \qquad \text{(Eq. SI 4)}$$

Having the reference matrix for each of the 102 possible models we can generate, each $\mathbf{M_r}(k)$ is converted into a one-hot encoding vector and used as output training for the neural network.

In the case of the time block, it is necessary to introduce a second input for the analysis and generate a correct output data set. For this purpose, we used the $\mathbf{M_r}(k)$ matrix (see Fig. SI 2). A vector of seven values was generated using the output label, in which each element is the time constant value of each decay pathway. Using the kinetic model shown in Eq. SI 4, we obtained the tau vector in a specific order:

$$model = \begin{bmatrix} -(k_1+k_2) & 0 & 0 & 0 \\ k_1 & -k_3 & 0 & 0 \\ k_2 & 0 & 0 & 0 \\ 0 & 0 & 0 & 0 \end{bmatrix} k_1 < k_2 \qquad \text{(Eq. SI 5)}$$

$$out_{tau} = \left[\frac{1}{k_1}, \frac{1}{k_2}, \frac{1}{k_3}, 0,0,0,0\right] \qquad \text{(Eq. SI 6)}$$

The amplitude block works similarly to the time block, which uses the $\mathbf{M_r}(k)$ matrix as a second input (see Fig. SI 2). However, the output has a 256 × 4 matrix in which each row is the spectrum of the corresponding electronic state (first row for A(t), second row for B(t), and so on).

Supplementary note 3: Evaluating DLRN analysis: model, time constants, and amplitude

Here, we compare the DLRN prediction to the expected results for some test data sets. Fig. SI 4 shows a comparison of DLRN-predicted models (red lines) and

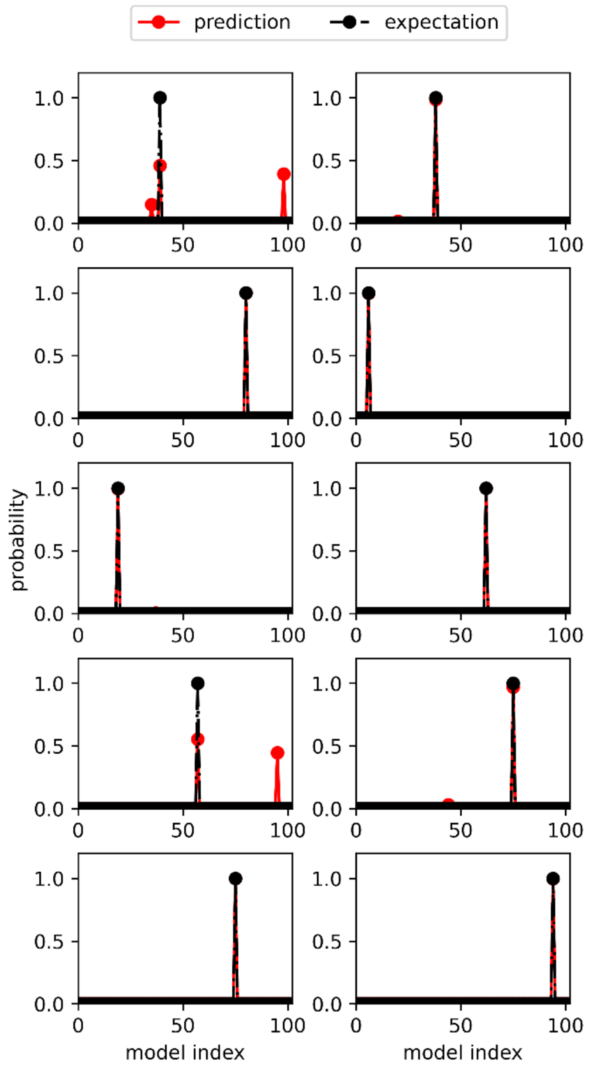

Fig. SI 4. Comparison of predicted (red lines) and expected (black dashed lines) model indexes for 10 different data sets. DLRN can well extrapolate the dynamics of systems, as also shown by the accuracy values in Table SI 1.

expected models (black dashed lines) for 10 random data sets. Specifically, the x position of each plot indicates the model index, which is linked to a specific concentration trace matrix **C**(t) of a kinetic model. The y axis represents the confidence probability. DLRN-predicted and expected models overlap excellently, with prediction confidence (y value) greater than 98% in almost all examples. A few cases showed multiple outputs from the DRLN analysis. However, at least one of the top 3 DLRN solutions matched the expected one, indicating that one possible solution is the exact model.

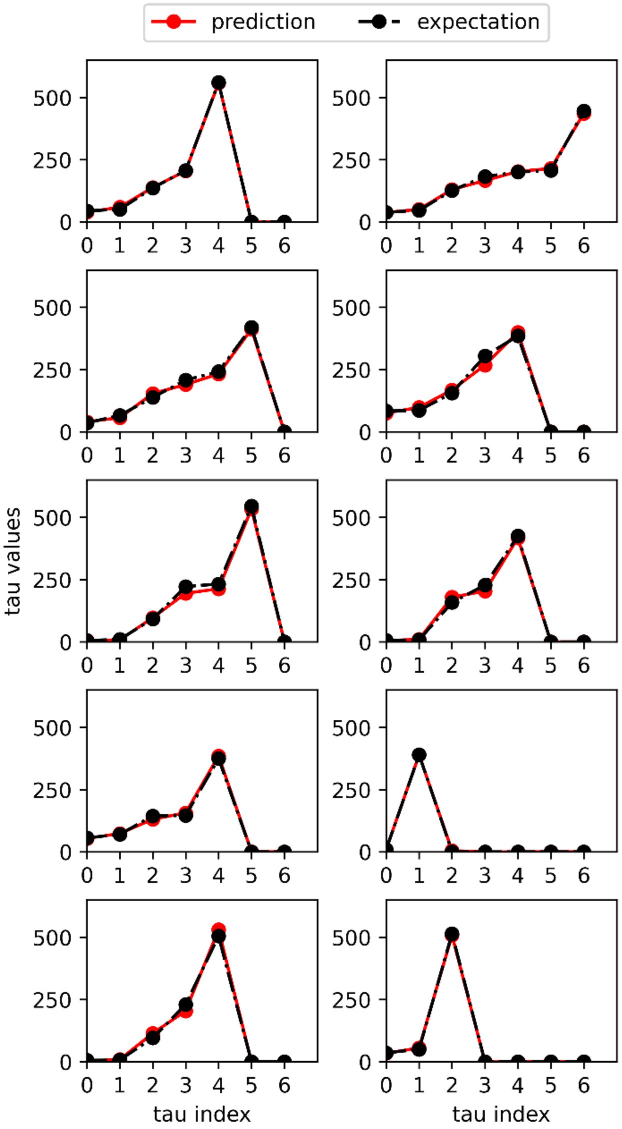

Fig. SI 5. Comparison of predicted (red lines) and expected (black dashed lines) time constants of specific pathways for 10 different data sets. DLRN analysis can extrapolate with good precision the number of time constants and their values. The accuracy of the DLRN, obtained using the area matrix, is shown in Table SI 1.

Fig. SI 5 shows a comparison of DLRN-predicted (red lines) and expected (black dashed lines) sorted time constant values for 10 different images. The x axis gives the index of each τ (index 0 = τ_1, index 1 = τ_2), while the y axis represents the corresponding time constants. The x axis has seven indexes (from 0 to 6) because each kinetic model can have a maximum of seven time constants due to the assumptions mentioned above. The plots in Fig. SI 4 demonstrate that DLRN predictions agree well with the expected curves, implying that DLRN can predict both the correct number of time constants and their corresponding values.

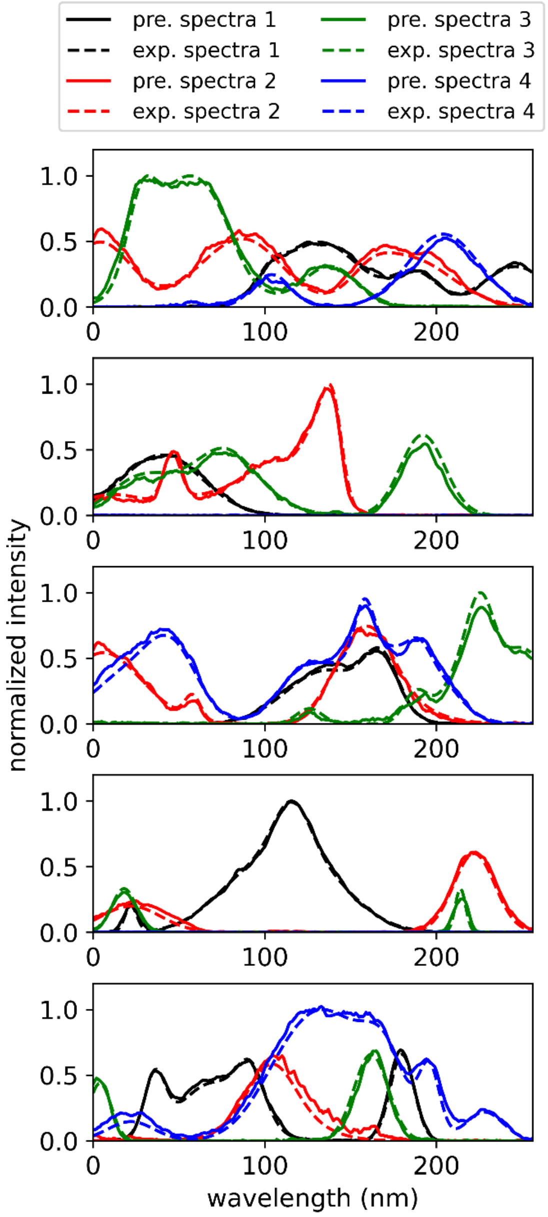

Fig. SI 6. Comparison of predicted (continuous lines) and expected (dashed lines) spectra of specific species for five data sets. DLRN can extrapolate the number of spectra (SAS) and their shapes with good approximation. The accuracy of the DLRN analysis is shown in Table 1 using the area metrics.

Fig. SI 6 shows a comparison between DLRN-predicted (continuous lines) and expected (dashed lines) spectral amplitudes for five different 2D datasets. In each graph, the x axis represents the wavelength, and the y axis shows the normalized intensity of the amplitudes (normalized by the image's maximum). The maximum number of DLRN amplitude predictions is four due to the method of generating the data (see "Generation of synthetic data for training and evaluation" in the Methods section). DLRN also predicted the correct spectra for complex structures with good approximation. Table SI 1 shows the accuracy values obtained for the model, time constants, and amplitude predictions during DLRN analysis on the evaluation batch. The area metric A_m (see Methods, main text) was used to quantify the accuracy of the regression analysis on the amplitudes and time constants. Starting with the model prediction on the evaluation batch, Table SI 1 shows the "Top 1" and "Top 3" accuracy values. Specifically, the Top 1 accuracy counts how often DLRN prediction and expectation have an exact match, while the Top 3 accuracy indicates when one of the three most probable predictions matches the ground truth. A detailed description of the evaluation analysis can be found in the section "DLRN analysis of synthetic time-resolved spectral data" in the main text.

Table SI 1. Accuracy of the predictions of kinetic model, time constants, and amplitudes from DLRN analysis of 100,000 2D data sets. A Top 3 accuracy value is also included for model prediction. Accuracy of time constants and amplitude calculated using the area metric A_M.

**Spectra predictions**	**Accuracy**		
	**Top 1**	**Top 3**	**A_M**
**Model**	82.0%	98.0%	–
**Time const. (A_M > 0.9)**	–	–	81.3%
**Time const. (A_M > 0.8)**	–	–	95.2%
**Amplitudes (A_M > 0.8)**	–	–	84.8%

Fig. SI 7 shows the comparison between DLRN predictions and expected results in the case when the initial state is a dark state, i.e., one that does not emit light but is involved in the dynamics. Whether all the components involved in the mechanism can be tracked depends on the experimental technique used to acquire the data. For

example, to track enzymatic reactions, a dye is activated only if the enzyme interacts with a specific molecule, resulting in a rising dye signal that indicates the formation of new molecules induced by the enzyme population. Fig. SI 6a shows the expected model with a relative time constant for each decay pathway compared to the DLRN prediction. The DLRN analysis showed good agreement with the expected kinetic model. Similar performance was obtained for the amplitude prediction, which slightly differed for the expected spectra (Fig. SI 6b). Notably, the amplitude for the initial state A is equal to zero, indicating that DLRN can understand when the initial state is “off”, but that it can also extrapolate the correct kinetic model. In this case, DLRN was also tested on an evaluation batch of 100,000 unkown 2D data sets. The results were similar to those shown in Table SI 1. An approximate difference of only 1–2% was observed in the predictions of amplitudes and time constants, which were 79,5% and 94% for the time constant prediction with area metrics greater than 0.8 and 0.9, respectively, whereas DLRN accuracy for the amplitude analysis was 79.7% for $A_M >$ 0.8. No significant changes were observed for the Top 1 and Top 3 model predictions, which showed similar accuracy values compared to the analysis performed on the batch dataset with initial state A “on”.

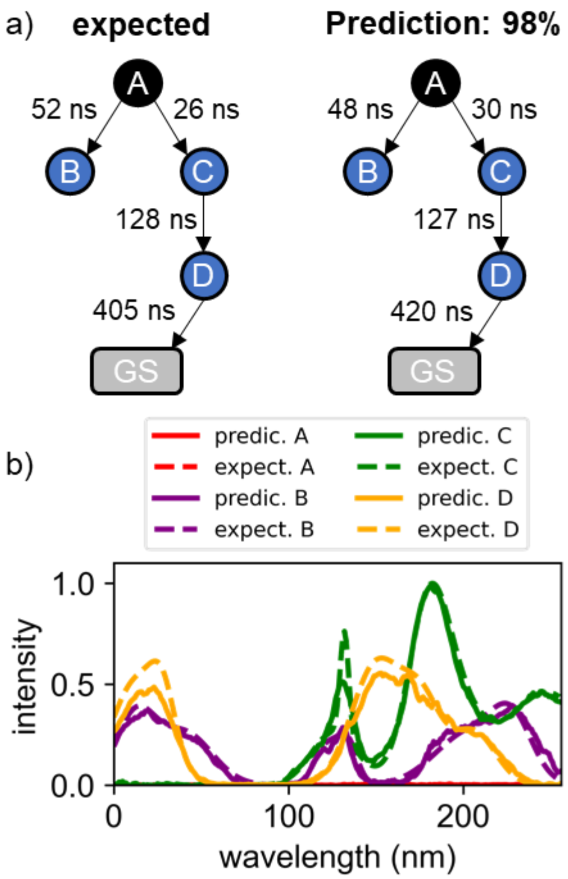

Fig. SI 7. DLRN analysis of systems having an initial dark state. a, Expected and DLRN-predicted kinetic models with the corresponding time constant for each pathway. Black circles indicate the dark state. **b,** Comparison between expected (dashed lines) and predicted (continues lines) spectra associated with the kinetic model analyzed. As expected, DLRN predicts a null amplitude for the first dark state A.

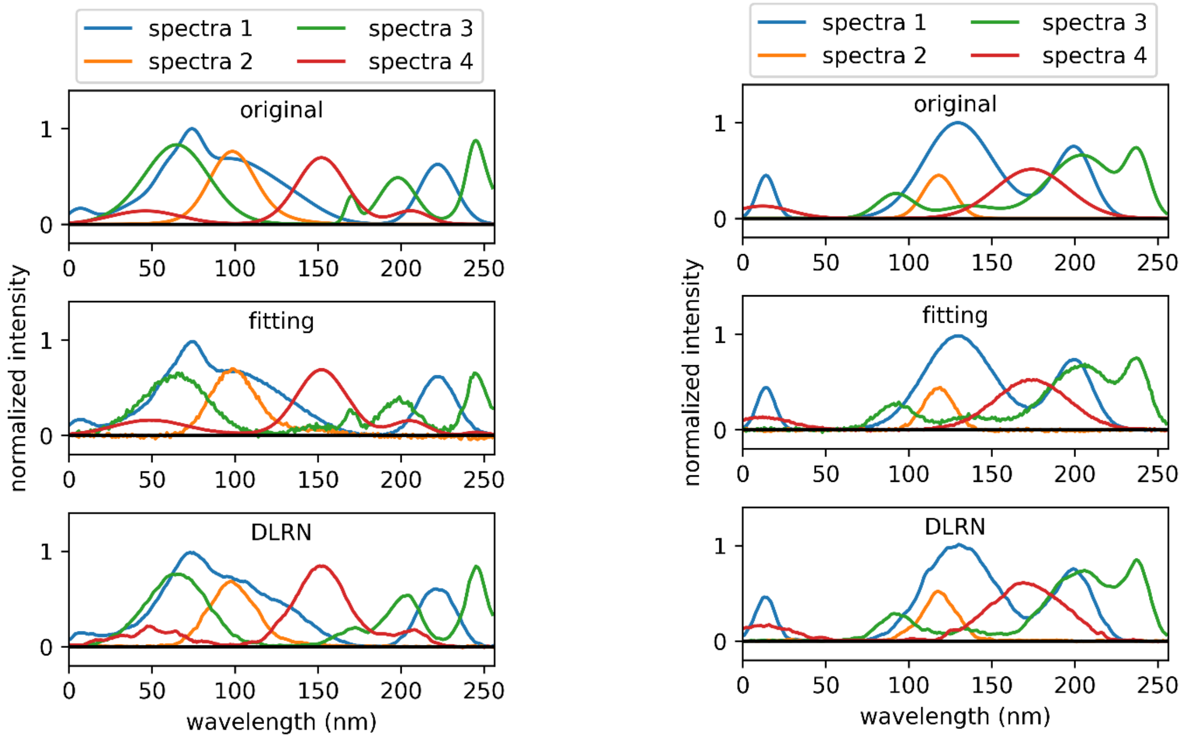

Fig. SI 8. Comparison of original spectra and the prediction from classical and DLRN analyses for two measurements. Classic and DLRN analyses show similar results, indicating that DLRN can perform spectral analysis well.

Fig. SI 8 shows the comparison between the expected spectra, the spectra obtained by fitting the data, and the DLRN analysis for two random data sets. In the main text, we show that DLRN sometimes performs better in cases of complex dynamics. However, Classical and DLRN analysis present very similar results for systems with more pronounced spectra, as shown in Fig. SI 8. Differences between expected (e) and predicted (p) spectra for ModiDeC, classical fit and kimopack from the analysis shows in Fig. 2 (man text) are presented in Fig. SI 9. These results support the efficiency and utility of DLRN in kinetic reaction networks analysis

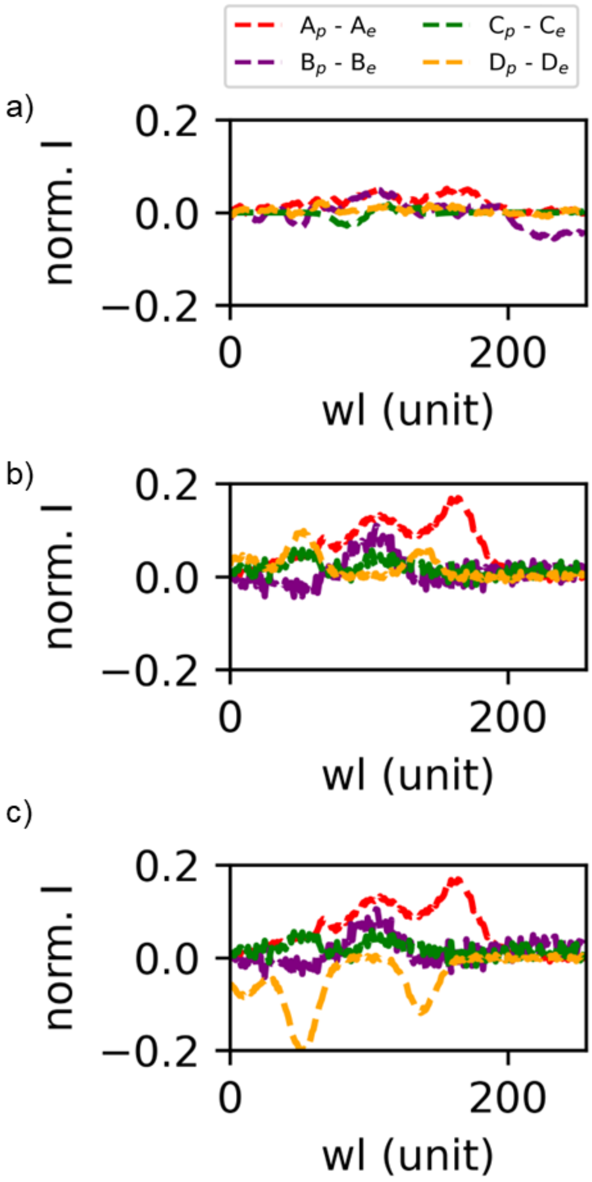

Fig. SI 9. a-c) difference between expected and predicted spectrum for DLRN (d), classical fit (e) and Kimopack (f). DLRN shows the less residuals in amplitude prediction.

Supplementary note 4: Selection of the area metrics limits

The area metric A_M was introduced to evaluate the accuracy of DLRN in predicting the values of the time constant and amplitude of a kinetic model. This metric requires a threshold value to determine whether a measurement can be considered positive or not (Methods, Area metrics for regression accuracy). For this study, we chose values of 0.8 and 0.9 for evaluating the time constant and a value of 0.8 for the amplitude prediction. The choice of these values can be explained using Fig. SI 10 as an example. The top graph shows a set of expected tau values (black line) and the area of values (blue area) within ± 20% error of the expected values. This area corresponds to all the possible values a DLRN prediction can have and yield a value of A_M greater than 0.8. By holding this value as a threshold for positive values, we consider all predictions to have an error on a single tau of less than 20%.

In the case of amplitude calculation, four spectra are predicted using DLRN. This means that the 20% error obtained using $A_M > 0.8$ must be divided for each spectrum, indicating an average error of 5%.

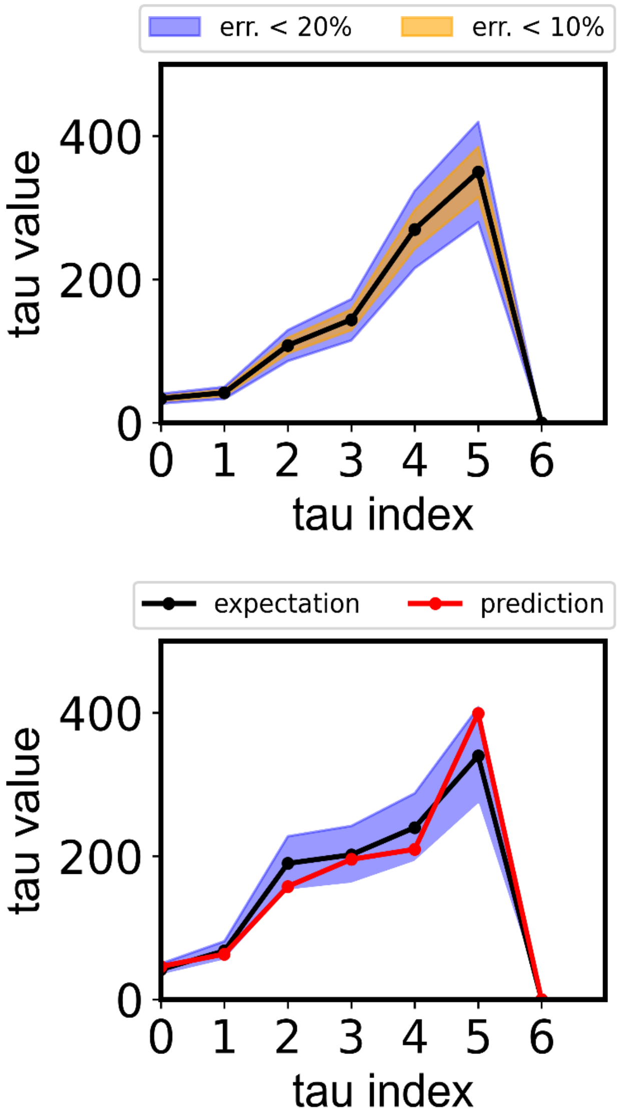

Fig. SI 10. Top: Set of time constants expectation value (black line). The blue area shows all the values within an error of 20% compared to the expectation. Bottom: Example of a DLRN prediction (red line) having A_m = 0.85 in comparison to the expectation. The blue area shows all the values within an error of 20% compared to the expectation.

Supplementary note 5: DLRN Analysis on time-resolved agarose gel

This section shows the model, time constants, and amplitude prediction on time-resolved agarose gel, as shown in the main text for the time-resolved spectra.

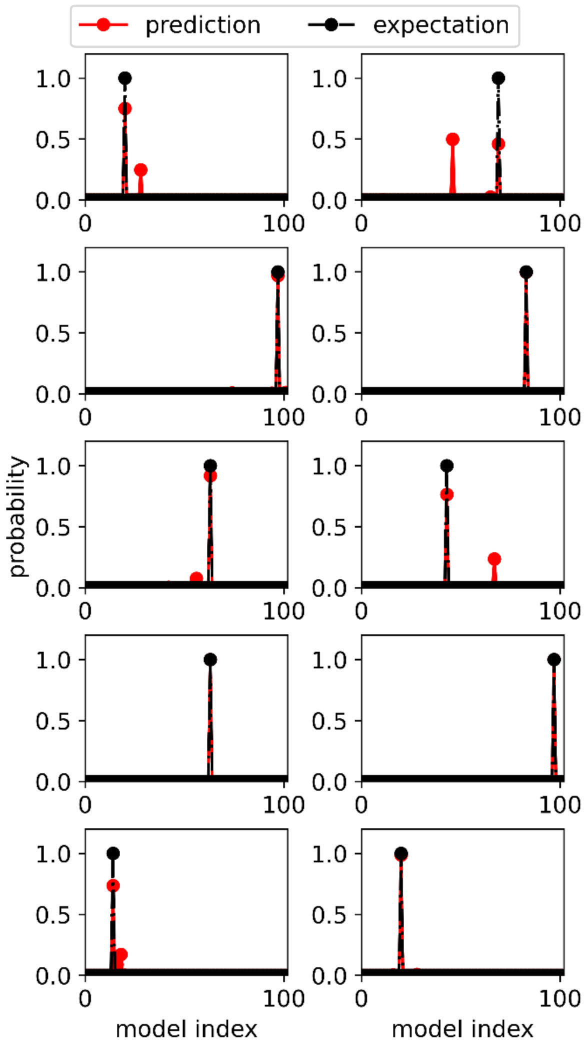

Fig. SI 11. Comparison of predicted (red lines) and expected (black dashed lines) model indexes for 10 different agarose gel images. DLRN can extrapolate the dynamics of systems well, as the accuracy values in Table SI 2 also show.

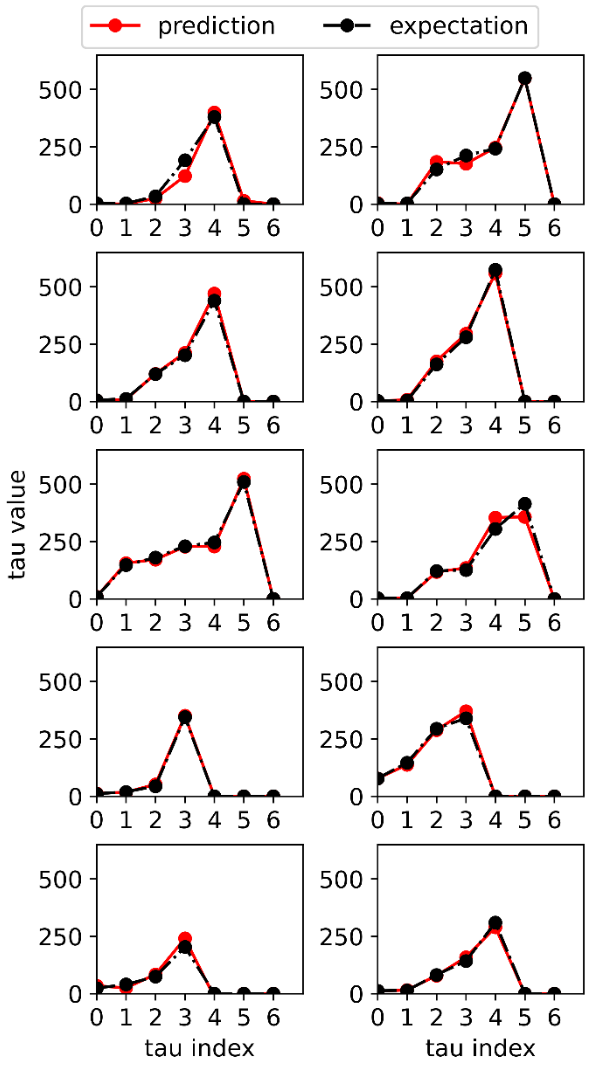

Fig. SI 12. Comparison of predicted (red lines) and expected (black dashed lines) time constant values of specific pathways for 10 different agarose gel images. DLRN can extrapolate with good precision the number of time constants and their value during the analysis. The accuracy of DLRN is shown in Table SI 2 using the area metric.

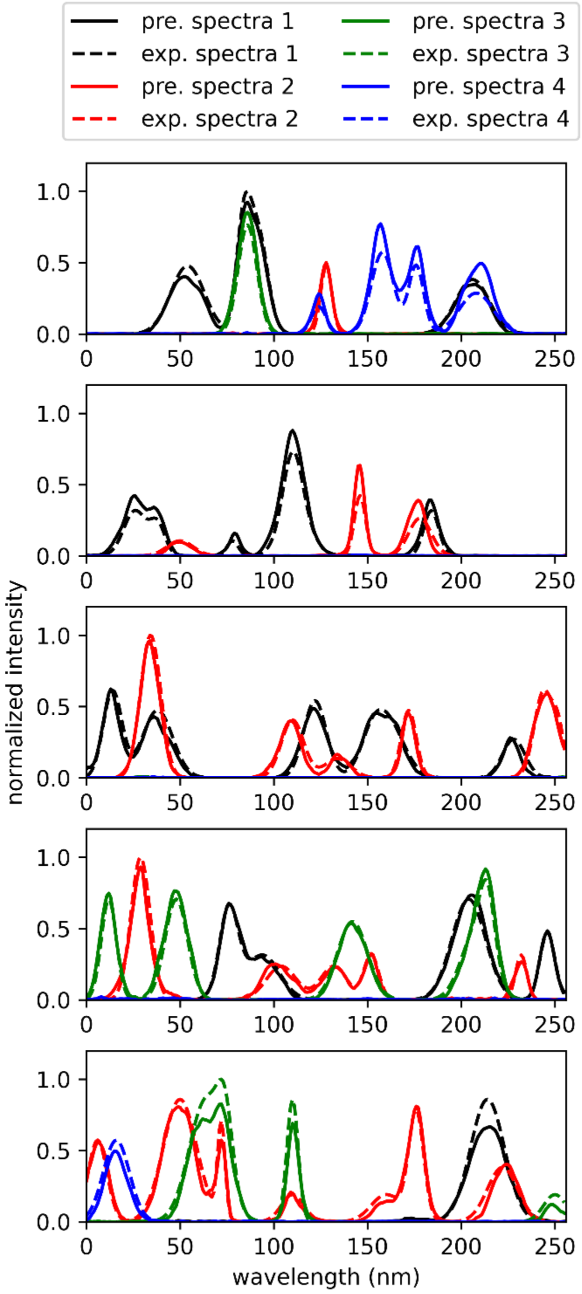

Fig. SI 13. Comparison of predicted (continuous lines) and expected (dashed lines) spectra of specific species for five agarose gel images. Each chart shows one data set. DLRN can extrapolate with good approximation the number of time spectra and their shapes. The accuracy of the DLRN analysis is shown in Table 2 using the area metrics.

Table SI 2 shows the accuracy values obtained for the model, time constants, and amplitude predictions during DLRN analysis on the evaluation batch. The area metric A_m (see Methods, main text) was used to quantify the accuracy of the regression analysis on the amplitudes and time constants. Table SI 2 shows the "Top 1" and "Top 3" accuracy values. Specifically, the Top 1 accuracy counts how often DLRN

prediction and expectation have an exact match, while the Top 3 accuracy indicates when one of the three most probable predictions matches the ground truth. A detailed description of the evaluation analysis can be found in the section “Analysis of synthetic time-resolved agarose gel electrophoresis using DLRN” in the main text.

Table SI 2. Accuracy of the predictions of kinetic model, time constants, and amplitudes from DLRN analysis of 100,000 2D data sets (agarose gels). A Top 3 accuracy value is also included for model prediction. Accuracy of time constants and amplitude calculated using the area metric A_M.

Agarose gel predictions	Accuracy		
	Top 1	Top 3	A_M
Model	82.4%	98.2%	–
Time const. (A_M > 0.9)	–	–	72.8%
Time const. (A_M > 0.8)	–	–	92.1%
Amplitudes (A_M > 0.7)	–	–	76.0%
Amplitudes + dilation filter (A_M > 0.7)	–	–	83.1%

Table SI 3. Accuracy of the predictions of kinetic model, time constants, and amplitudes from DLRN analysis of 100,000 2D data sets (transient absorption). A Top 3 accuracy value is also included for model prediction. Accuracy of time constants and amplitude calculated using the area metric A_M.

Transient Absorption	Accuracy		
	Top 1	Top 3	A_M
Model	87.0%	98.5%	–
Time const. (A_M > 0.9)	–	–	81.7%
Time const. (A_M > 0.8)	–	–	96.6%
Amplitudes (A_M > 0.8)	–	–	83.2%

Table SI 4. R^2 values between DLRN predictions and expected values for time constants and amplitudes for transient absorption (TA) photoluminescence (PL) agarose gel (Gel). 100,000 2D data sets were used for analysis.

	TA	TA	PL	PL	Gel	Gel
	Time const.	Amplitude	Time const.	Amplitude	Time const.	Amplitude
R^2 values	98.0%	95.5%	98.1%	95.0%	96.9%	92.0%

Supplementary note 6: DNA strand displacement: additional analysis

Fig. SI 14 shows the expected and forced kinetic traces of the two DSD kinetic reactions shown in Fig. 5 of the main text. Forced kinetic traces were obtained by using a combination of DLRN predictions (for $\tau_{A\to B}$ and $\tau_{A\to C}$) and calculated (for $\tau_{C\to D}$ and $\tau_{B\to D}$) tau values. The graph shows that expected and forced kinetic traces diverge significantly when the calculated $\tau_{C\to D}$ and $\tau_{B\to D}$ are used, pointing that DLRN analysis was correct.

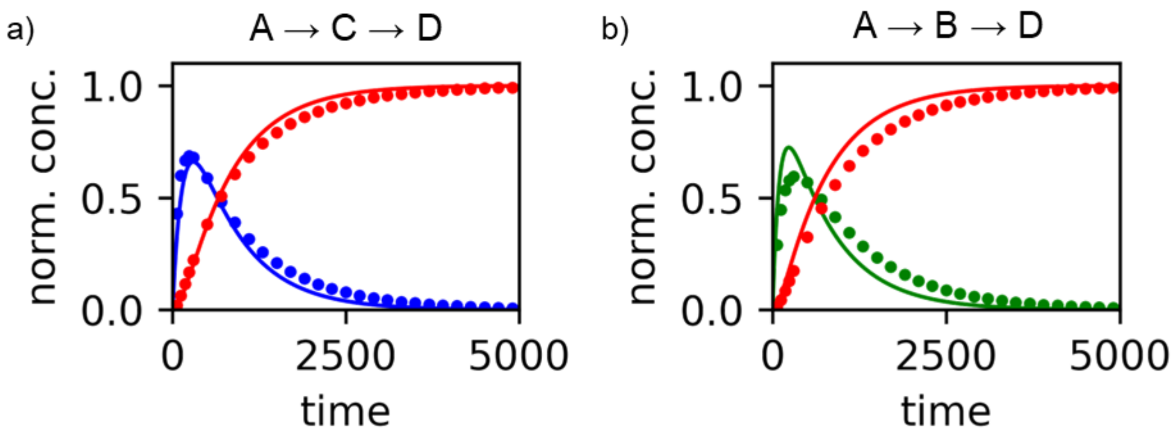

Fig. SI 14. Expected (circles) and forced (continuous lines) kinetic traces for the state C (blue), B (green), and D (red) for DSD kinetic reaction A→C→D (a) and A→B→D (b). The forced kinetic traces are a combination of DLRN predictions for the initial reaction (A→C and A→B), whereas for the second reaction step (C→D and B→D) the expected value of 721 s was used. The predicted kinetic traces differ significantly from the expected values, suggesting that DLRN tau predictions were correct.

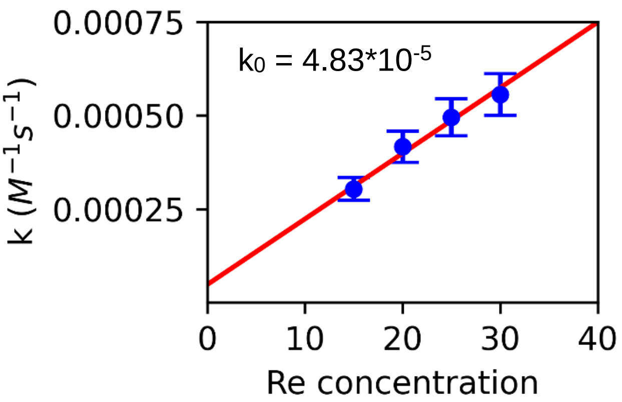

Fig. SI 15. Rate constant $k_{C\to D}$ predicted with DLRN for different concentration values of Re. Imposing an error of 10% (DLRN maximum error), the linear fit gives an intercept value (k_0) of 4.83×10^{-5}, which is similar of the expected rate constant of 4.62×10^{-5}. The difference in these two values is approximately 4.5%.

Fig. SI 15 shows the rate constants for C→D kinetic pathways obtained from DLRN analysis using four Re concentrations and then fitted by a linear regression. The intercept represents the value with no Re present, which can be compared to the theoretical value of $k_0 = 4.83 \times 10^{-5}$. The linear regression shows an intercept value of 4.62×10^{-5}, which is only 4.5% different than k_0. Fig. SI 16a shows the residuals obtained by DLRN analysis of the full DSD dynamics if both Su1 and Su2 are present. The errors of the residuals were not significant, indicating the predictions from the DLRN were good. Fig. SI 16b presents the normalized kinetic traces for states B and C, showing the remarkably similar dynamics of the two states and demonstrating that disentangling the two contributions is extremely difficult.

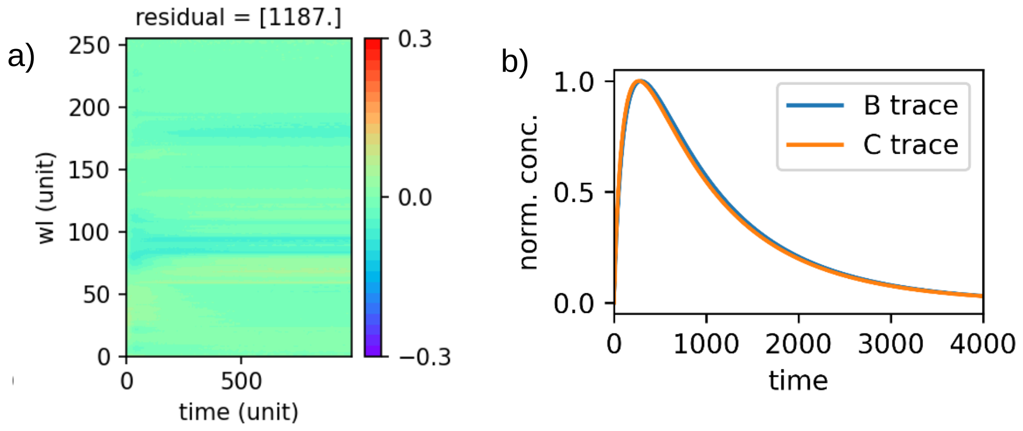

Fig. SI 16. a, Residuals obtained from DLRN analysis. **b,** Expected kinetic traces for the states B and C (see Fig. 6 in the main text) normalized by their respective maximum values. The two traces decay with the same time constants, making it difficult to disentangle the dynamics and ascertain the correct amplitude for the two states.

Fig. SI 17 shows the results of classical fitting on the DSD full dynamics. To fit the data, the following set of differential equations was used as a model:

$$\begin{bmatrix} dA/dt \\ dB/dt \\ dC/dt \\ dD/dt \end{bmatrix} = \begin{bmatrix} -k_1 & 0 & 0 & 0 \\ \alpha * k_1 & -k_2 & 0 & 0 \\ (1-\alpha) * k_1 & 0 & -k_3 & 0 \\ 0 & k_2 & k_3 & 0 \end{bmatrix} \begin{bmatrix} A \\ B \\ C \\ D \end{bmatrix} \quad \text{(Eq. SI 7)}$$

where alpha is a fitting coefficient that can vary between 0.01 and 1. Here, fitting gave an alpha value of 0.26. Despite the use of the correct hypothetical model for the fit, the classical fitting yielded worse results than DLRN, albeit with slightly cleaner amplitude values.

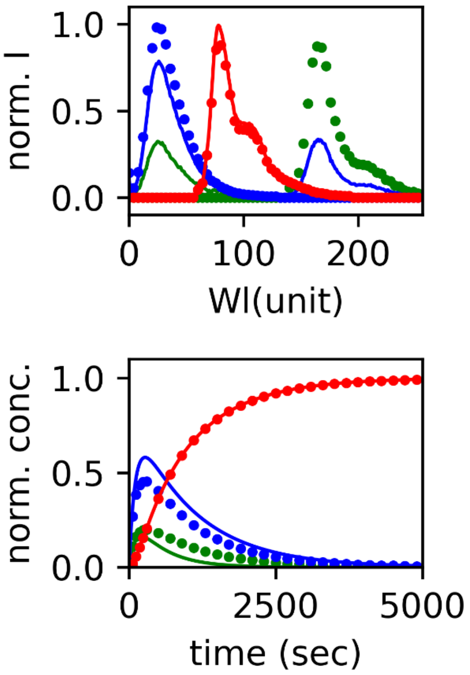

Fig. SI 17. Expected (circles) and classically fitted (continuous lines) amplitudes (top) and kinetic traces (bottom) for the branching kinetic reaction (Fig. 6 in the main text). The colors correspond to the states in Fig. 6. To fit the data, a branching mechanism and only positive amplitudes were imposed. Surprisingly, with respect to the kinetic traces the fitting performed worse than DLRN, but a cleaner amplitude prediction was obtained. However, the expected and predicted spectra for state B (green) significantly differ.

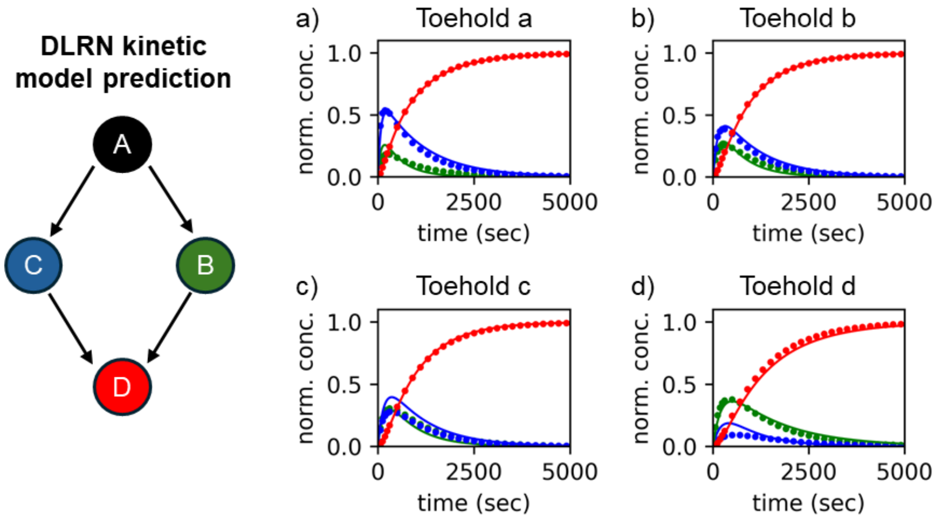

Fig. SI 18. Left: DLRN kinetic model prediction obtained by analyzing the four CRN variants having toeholds a–d. Right: Expected (circles) and DLRN-predicted

(continuous lines) kinetic traces if toeholds a, b, c, and d (a–d, respectively) were used. Colors correspond to the kinetic model.

Supplementary note 7: G-User-DLRN: a graphic user interface for DLRN analysis

This section provides a protocol for using the DLRN GUI for data analysis. The steps for running the GUI after starting the program called "DLRN_GUI" are given below. A new window appears from which a number of options can be selected (Fig. SI 19a).

1. Select Spectra or Agarose Gel from the first set of options. This will load the pretrained DLRN model for the respective scenario. This can take a few minutes.
2. Select Top 1 or Top 3 from the second set of options. This will change the analysis output, giving the solution for either the most probable output or the three most probable outputs.
3. Select the scale factor (suggested value = 1). This rescales the timescale to let DLRN analyze data sets with a time window larger than one timescale. However, using a large value for the scale factor can change the results of the analysis due to data interpolation during preprocessing data preparation.
4. Load the timescale to be used for the measurements. It is important to rescale the data with one that matches the timescale used during the DLRN analysis.
5. Load the data to be analyzed using "load the data" (located at the bottom). Search for the data that you want to analyze using the browser window. Only a NumPy zip file (.npz) having a subfolder "train" or .txt/.dat files can be loaded in the GUI.
6. (Optional) Is it possible to test the DLRN performance using the "Test DLRN" button. This allows the user to try a few ground truth data to check the performance.
7. Click the "Data Analysis" button to start the analysis and obtain the DLRN analysis results. This can be done only after the compulsory steps (1–5) have been completed. A typical output of the analysis is shown in Fig. SI 19b–d.

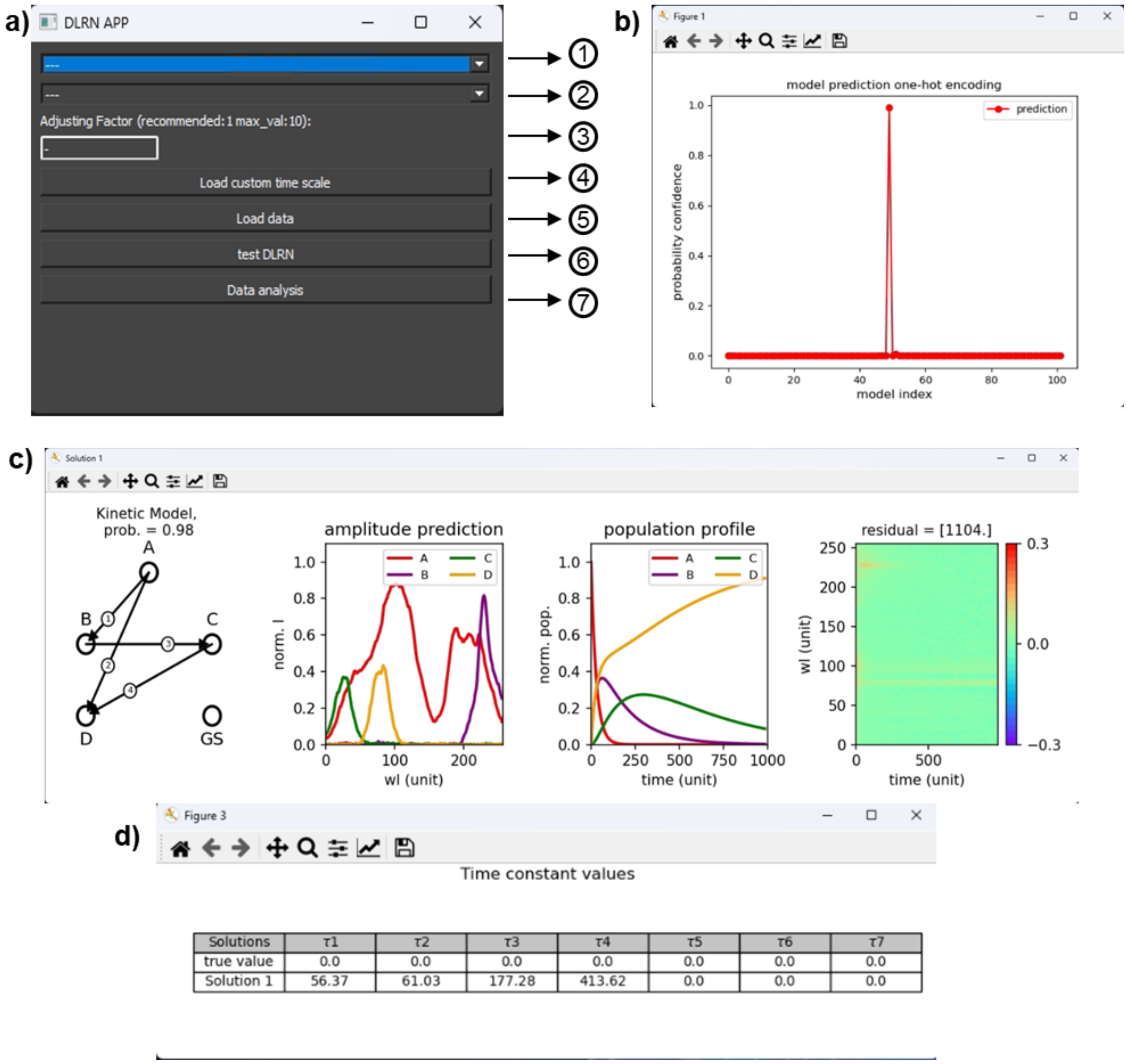

Fig. SI 19. a, The G-user-DLRN interface. The options 1–7 are outlined in the instructions provided above. **b–d,** The results obtained by DLRN analysis using the Top 1 solution: the one-hot encoding representation (b); the predicted kinetic model, tau prediction, and so forth (c); the table of tau values (d). If Top 3 analysis is selected, three windows like that shown in panel c appear, each detailing the DLRN results for their respective solution.

Supplementary note 8: DNA sequences and corresponding reaction rates in the DSD

We calculated the binding rate coefficients for toeholds (k_{bind}) using Eq. SI 10, with equilibrium concentrations obtained from NUPACK, assuming $k_{\text{unbind}} = 0.012\ \text{s}^{-1}$, $[Na^+]$ = 100 mM, $[Mg^{2+}]$ = 10 mM, and 37 °C.

$$\text{t1} + \text{t1}^* \rightleftharpoons \text{t1t1}^* \qquad \text{(Eq. SI 8)}$$

$$K_{eq} = \frac{k_{\text{bind}}}{k_{\text{unbind}}} = \frac{[\text{t1t1}^*]}{[\text{t1}][\text{t1}^*]} \quad \text{(Eq. SI 9)}$$

$$k_{\text{bind}} = \frac{[\text{t1t1}^*]}{[\text{t1}][\text{t1}^*]} k_{\text{unbind}} \quad \text{(Eq. SI 10)}$$

However, this estimation does not take into account the significant effect of fluorophores and quenchers. The results of preliminary experiments have suggested that the real processes are much faster, therefore we used 100-fold higher values for k_{bind}. The values are summarized in Table SI 4.

Table SI 3. Realistic DNA sequences for the above DNA reaction network.

Name	Structure	Sequence
Inp	1 2 3 4	a: AATAATCTACC TCAGCACATCGTATCAAACTCGTCCATGGGTTAGG<br>b: CTAATCTACCTCAGCACATCGTATCAAACTCGTCCATGGGTTAGG<br>c: GCTCTACCTCAGCACATCGTATCAAACTCGTCCATGGGTTAGG<br>d: AATCTACCTCAGCACATCGTATCAAACTCGTCCATGGGTTAGG
Su1	2 5 3 6<br>6*3*2*1*	TCAGCACATCCTGTTCGGTATCAAACTCGTCCAT\Atto425\TGTC<br>a: GACA\Q\ATGGACGAGTTTGATACGATGTGCTGAGGTAGATTATT<br>b: GACA\Q\ATGGACGAGTTTGATACGATGTGCTGAGGTAGATTAG<br>c: GACA\Q\ATGGACGAGTTTGATACGATGTGCTGAGGTAGAGC<br>d: GACA\Q\ATGGACGAGTTTGATACGATGTGCTGAGGTAGATT
Su2	7 2 5 3<br>8*4*3*2*7*	ATAGTCAGCACATCCTGTTCGGTATCAAACTCGTCCAT\Cy5.5\<br>CCTAACCC\Q\ATGGACGAGTTTGATACGATGTGCTGACTAT
Re	3<br>3*5*	GTATCAAACTCGTCCAT\Cy3\<br>\Q\ATGGACGAGTTTGATACCGAACAG

Q indicates a wide-range quencher. The spectra used in the analysis were generated according to the fluorophores used: Atto425, Cy5.5, and Cy3.

Table SI 4. Binding rate coefficients for the different toeholds.

Toehold name	Sequence	k_{bind} ($nM^{-1}s^{-1}$)
a	AATAATCTACC	1.43×10^{-3}
b	CTAATCTACC	5.73×10^{-4}
c	GCTCTACC	2.94×10^{-4}
d	AATCTACC	5.58×10^{-5}
4	GGGTTAGG	3.29×10^{-4}
5	CTGTTCG	4.62×10^{-5}

Supplementary note 9: Expanding DLRN analysis: multi-time scale and complex model analysis

In this section, we show a second multi-time scale and complex model analyzed by DLRN.

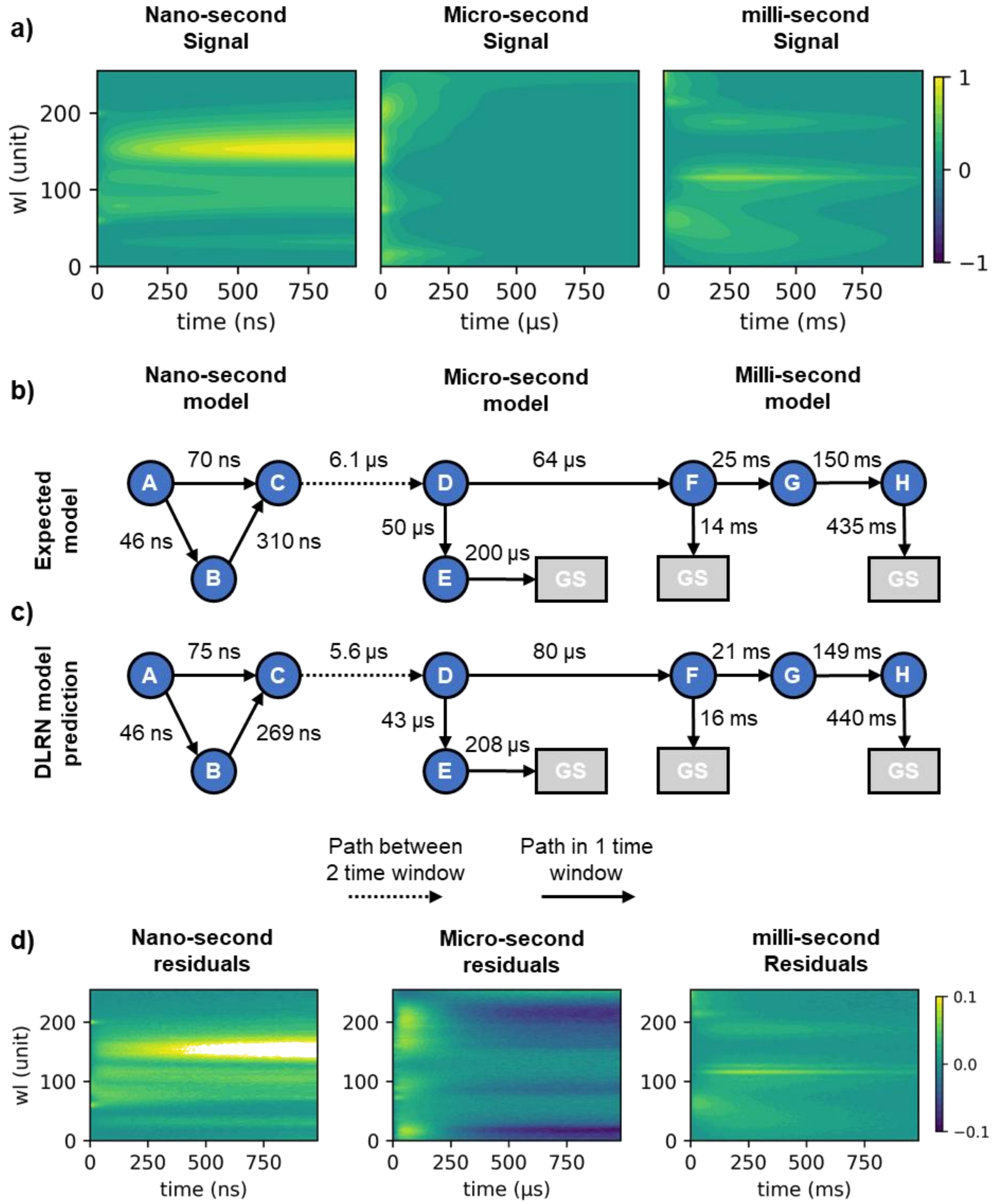

Fig. SI 20. a) Nanosecond (left), microsecond (middle) and millisecond (right) time resolved signal analyzed with DLRN. b) expected kinetic model with decay time constants for each pathway. c) predicted kinetic model using DLRN. predicted time constants are shown for each decay pathway. d) residual between original signal and DLRN fit.

Similar to what observed in Fig. 3 in the main text, using DLRN we were able to reconstruct the complex dynamics of the systems by analyzing individual each of the three time scales. The final DLRN model was an interpretation and recombination of the three results. Nano-second low residual values are associated to the transition species (decays of C). Low time points representation of a pathway usually affects time or amplitude extraction also for classical fit methods.

Supplementary note 10: Testing DLRN with variable testing datasets: granularity and noise resistance of the neural network

In this section, we want to test DLRN in scenarios that were different compared to the training dataset. We would like to test if time scale and noise can affect the neural network performance. Moreover, we want to understand the limit condition where DLRN could be used for the data analysis. Figure SI 21 shows DLRN performance on 5 different Δt values for model, time constants and amplitude predictions. In detail, we used a linear scale with different Δt. For each Δt, 20k datasets were created for the analysis. The analysis showed that model, time constants and amplitudes prediction remain quite consistent for $\Delta t \leq 2$, while DLRN performances reduction start to be visible for $\Delta t = 10$ (performances reduction of about 15%, see blue Bar Fig. SI 21). The orange bars are less affected, with a performance reduction of about 8%. Drastic reductions were observed for $\Delta t = 20$. We associate the reduction in DLRN performance with the fact that a large part of the initial dynamics is lost during interpolation when large Δt is used. This is because DLRN is trained with a combination of linear (for initial dynamics) + exponential (for long dynamics) time scale, which means that large Δt will not represent the initial dynamics but only the long one.

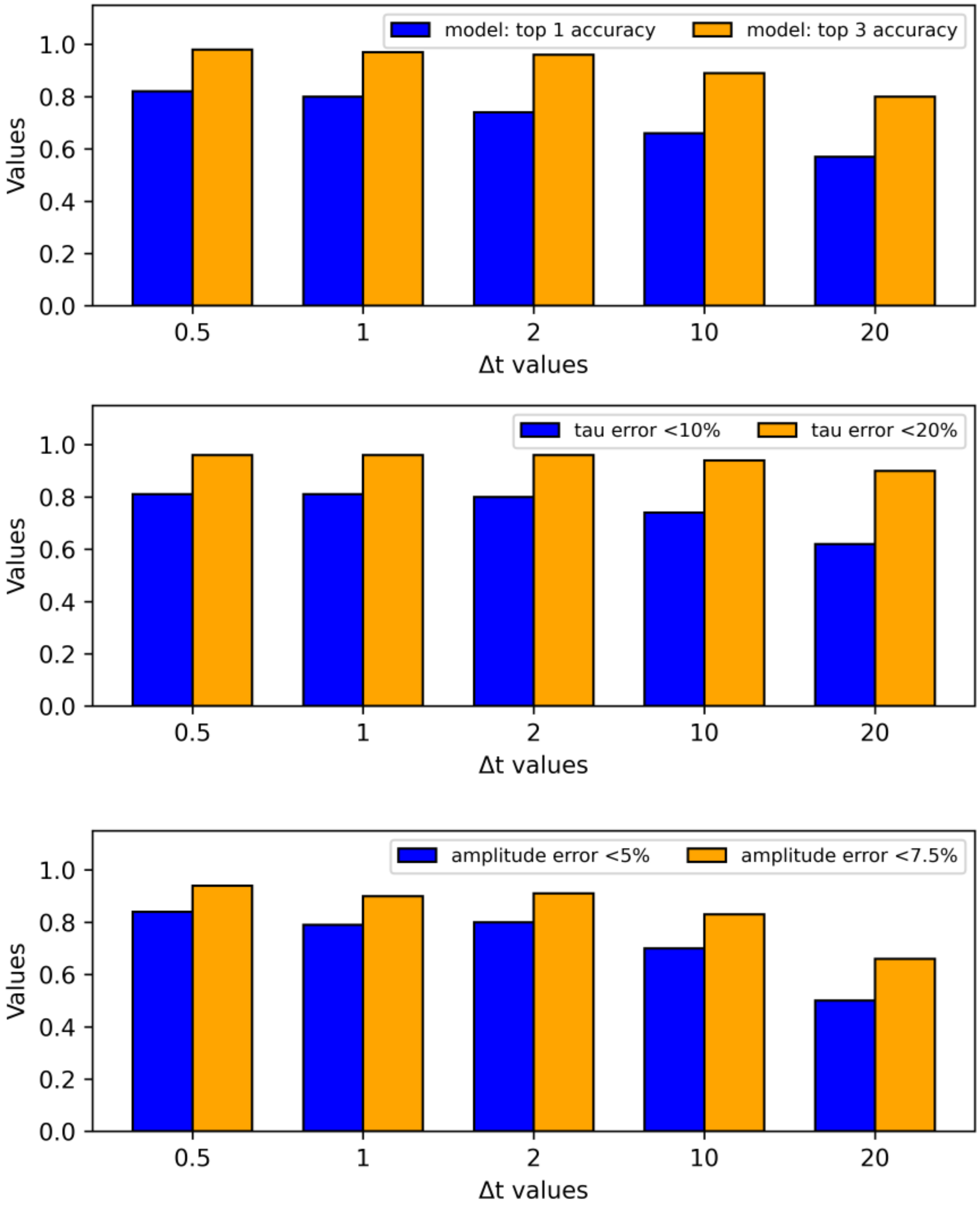

Fig. SI 21. DLRN model (top), time constants values (middle) and amplitudes values (bottom) prediction performances for several time step (Δt) using a linear scale. The time scale is always interpolated back to the time scale used for the training. Granularity can affect the neural network performance, especially because initial dynamics can be lost during the interpolation when Δt is large.

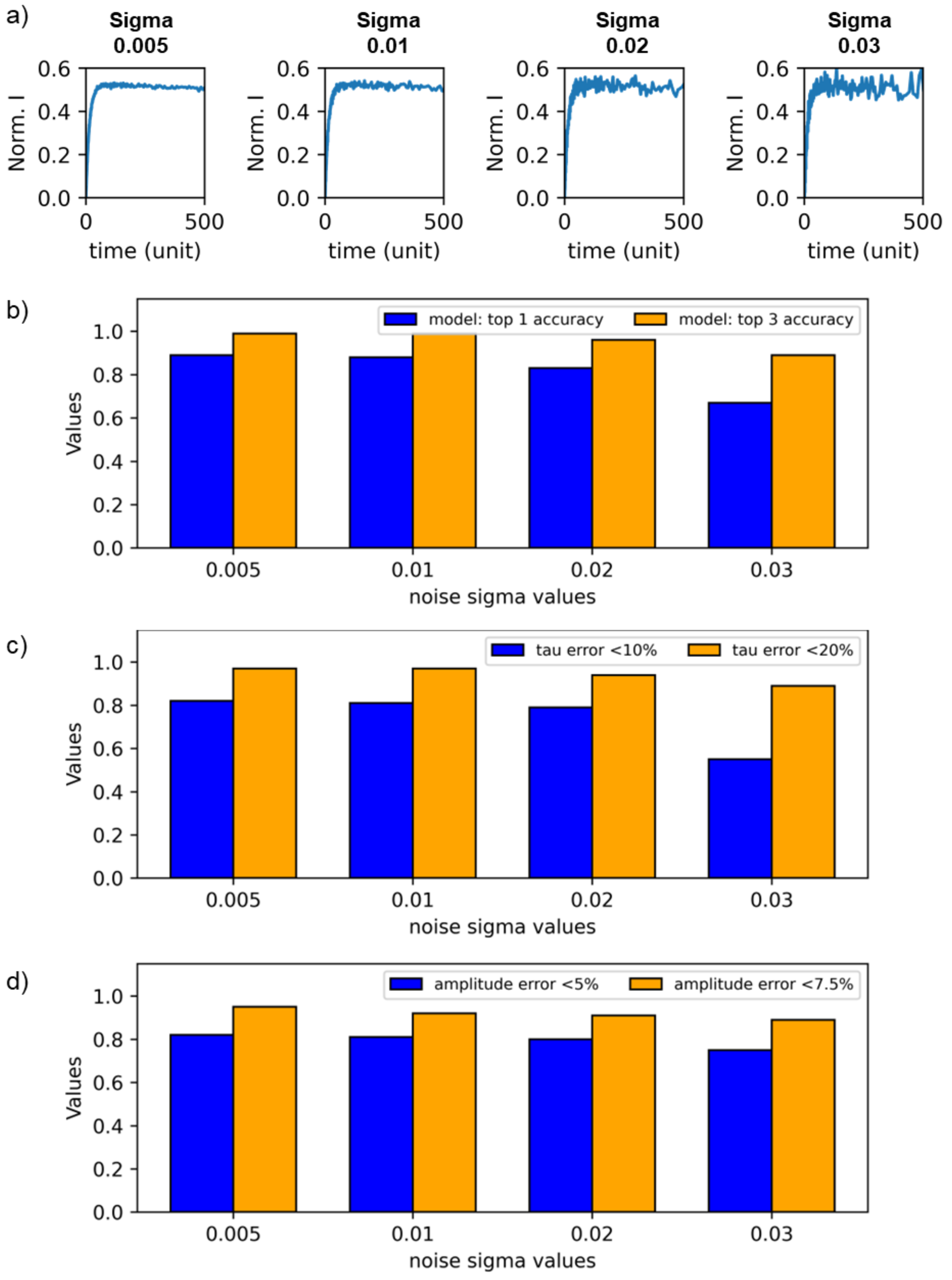

Fig. SI 22. a. Same kinetic trace with different noise sigma value analyzed in this work. Already to a value of 0.03 it is possible to observe a severe noise effect. b-d) DLRN model (b), time constants values (c) and amplitudes values (d) prediction performances for several noise sigma values using a random normal distribution.

Supplementary note 11: Comparing transient absorption DLRN results with existing fitting methods

In this section, KimoPack and DLRN analysis on a linear (Fig. SI 23) and branched (Fig. SI 24) mechanisms are shown. Both algorithms perform well in the linear case, while only DLRN seems to analyze correctly the branching mechanism. Moreover, DLRN alone predicts the kinetic model, while in the case of KimoPack it is necessary to specify the kinetic model before the analysis.

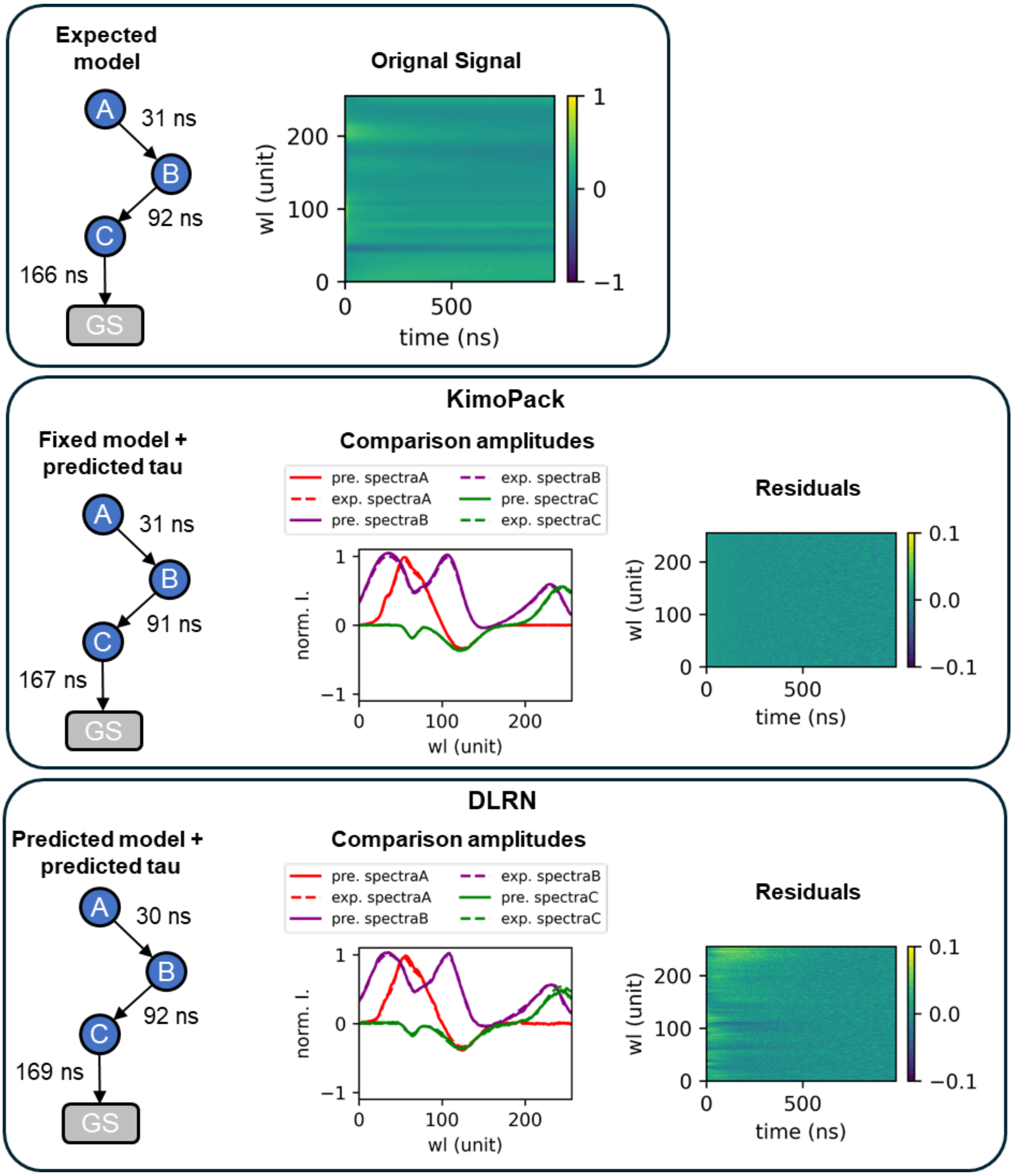

Fig. SI 23. Top. Expected kinetic model with time constants for each decay pathway. The normalized transient absorption (TA) signal is shown as well. **Middle.** KimoPack analysis on the normalized TA signal. Fixing the model, it was possible to obtain the correct time constants and amplitudes values with good residuals. **Bottom.** DLRN analysis on the normalized TA signal. DLRN predicted correctly the kinetic model, showing a linear reaction with correct decay time constants and amplitudes, including acceptable residuals.

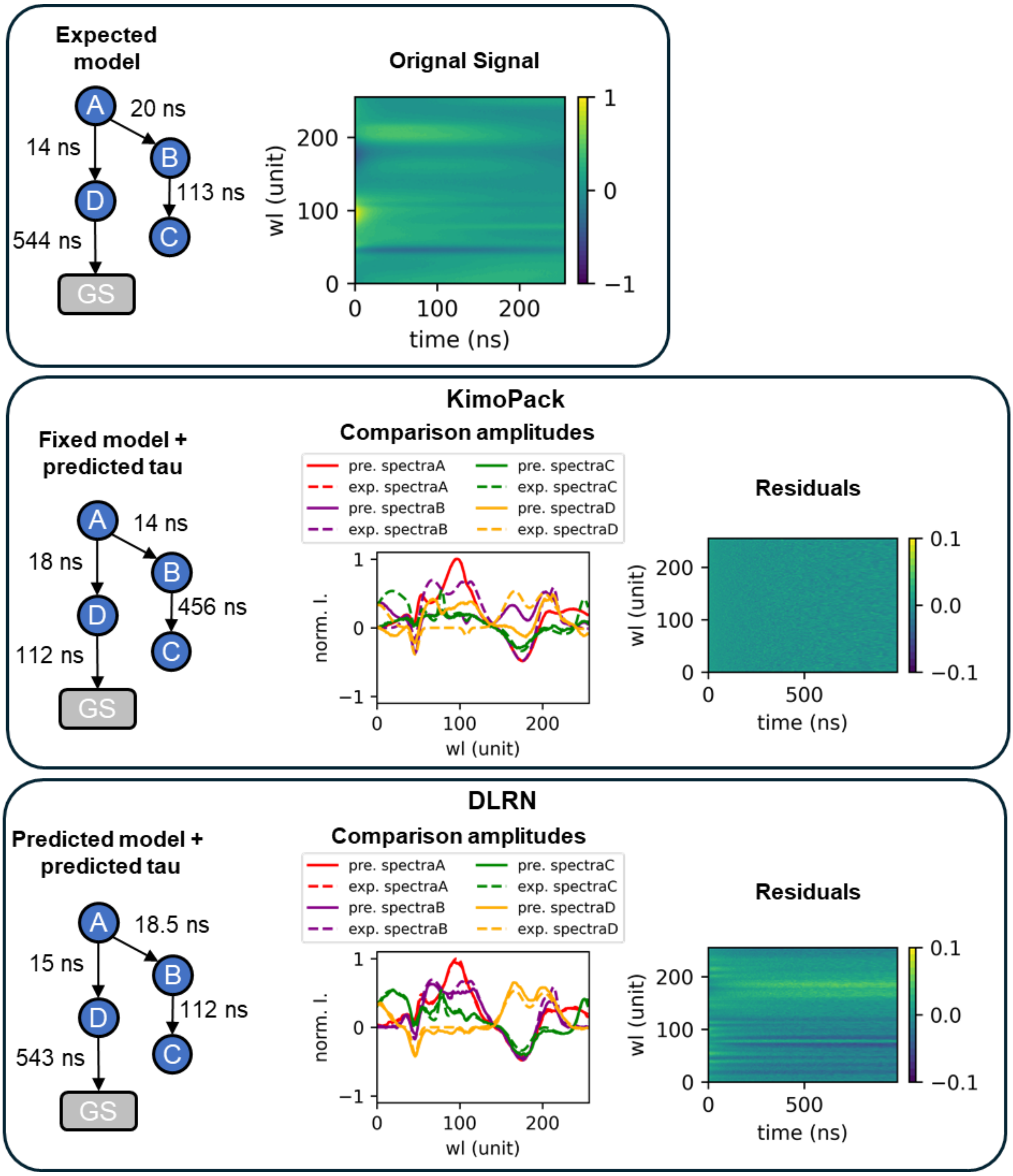

Fig. SI 24. Top. Expected kinetic model with time constants for each decay pathway. The normalized transient absorption (TA) signal is shown as well. **Middle.** KimoPack analysis on the normalized TA signal. Fixing the model, it was not possible to obtain the correct time constants and amplitudes values, despite good residuals were shown. For example, D and B decay time constants shows significant difference compared to the expected values. **Bottom.** DLRN analysis on the normalized TA signal. DLRN predicted correctly the kinetic model, showing a complex branching reaction with correct decay time constants and amplitudes, including acceptable residuals.
